# Recent advances in targeting cyanine dyes: hierarchical targeting mechanisms and theranostic integration for multi-disease precision therapy

**DOI:** 10.7150/thno.132719

**Published:** 2026-06-25

**Authors:** Shuai Zhang, Yang Xu, Yu Wang, Zelin Chen, Yang Wang, Quanying Liu, Tantan Wang, Yali Dai, Chunmeng Shi

**Affiliations:** Institute of Rocket Force Medicine, State Key Laboratory of Trauma and Chemical Poisoning, College of Preventive Medicine, Army Medical University (Third Military Medical University), Chongqing 400038, China.

**Keywords:** near-infrared fluorescence imaging, targeting cyanine dyes, heptamethine cyanine dyes, theranostic integration, precision disease therapy

## Abstract

The development of novel theranostic agents is a key initiative to address current limitations in disease diagnosis and therapy. Notably, targeting cyanine dyes (TCDs), by virtue of their excellent optical imaging performance, versatile structural modifiability, and multi-dimensional targeting specificity, facilitate the directional recognition of disease regions and exhibit tremendous application potential in theranostics. While a variety of TCDs have been successfully developed and their theranostic efficacy experimentally validated, researchers still lack a systematic summary of related studies. Although related advances in fluorescent probes, tumor theranostics, and NIR-II fluorophores have been reviewed, a focused and systematic overview of TCDs in terms of their synthesis, hierarchical targeting mechanisms, and multi-disease theranostic applications remains limited. Accordingly, this article systematically reviews TCDs’ synthesis strategies, elucidates their “tissue-cell-organelle” hierarchical targeting mechanism, and summarizes the therapeutic applications in diseases including tumors, fibrotic diseases, metabolic diseases, and radiation-induced injuries. Compared with previous reviews, this review highlights the structure-inherent targeting properties of TCDs, their hierarchical targeting mechanisms, and their emerging theranostic potential beyond oncology. Meanwhile, this article outlines the core advantages and current challenges of TCDs in theranostic integration, and delineates key future directions, including precise and intelligent molecular design, photostability limitations, systematic preclinical evaluation, multimodal technology integration, and the expansion of disease application scenarios. It aims to provide comprehensive theoretical support for advancing the fundamental research and clinical translation of TCDs.

## 1. Introduction

Conventional diagnostic and therapeutic approaches have many limitations in practical application 1, 2, including insufficient specificity 3-5, unsatisfactory therapeutic effects 6, and prominent adverse reactions 7, 8. Accordingly, improving the overall efficiency of disease detection and treatment remains a major challenge in biomedicine. Therefore, developing novel diagnostic and therapeutic tools with high targeting ability and multi-modal functionality has become the key to overcoming the current challenges in diagnosis and treatment 9.

Cyanine dyes, as a class of small-molecule compounds with unique optical and chemical properties, offer a novel technical approach to addressing the aforementioned theranostic challenges. Cyanine dyes include multiple subtypes, with heptamethine cyanine dye and hemicyanine dye being the two mainstream categories. Their core structure consists of two heterocyclic rings connected by a π-conjugated polymethine chain, and the diverse combinations of heterocyclic ring types and side-chain substituents provide ample opportunities to regulate optical properties 10-13. These dyes typically exhibit near-infrared (NIR) absorption and emission wavelengths (700-1000 nm), allowing effective tissue penetration, reduced autofluorescence interference, and high sensitivity for *in vivo* imaging 14. Traditionally, cyanine dyes (such as indocyanine green, ICG) have been recognized as non-targeting agents. Since 2008, our group and collaborators first reported the preferential accumulation of IR-780 dye and its derivatives in malignant tissues through an intrinsic targeting mechanism without any structural modification, targeting ligand, or nanocarrier, providing a structure-inherent active targeting modality for medical imaging and theranostics 15. This unexpected finding suggested a top-down strategy and upended the prevailing bottom-up strategy that selective drug delivery requires molecular complexity, and was subsequently validated by independent laboratories worldwide. To clearly define such targeting functional materials, this review refers to cyanine dyes with actively structure-inherent targeting recognition capabilities as targeting cyanine dyes (TCDs).

The multi-dimensional targeting capabilities of TCDs exhibit distinct hierarchical characteristics, which can achieve precise targeting recognition at the tissue, cell, and subcellular levels 16-18. Specifically, this targeting function can be achieved through different pathways: regulating the hydrophilicity/hydrophobicity, charge state of molecules, or utilizing inherent pathological microenvironment features such as hypoxia and high glycolysis, and each level of targeting is closely associated with theranostic applications. At the tissue, TCDs accumulate in pathological sites stress and energy-dependent via the enhanced permeability and retention (EPR) effect or specific ligand-receptor interactions 19, 20. Moving to the cellular, transmembrane transport mediated by organic anion transporting polypeptides (OATPs) and albumin-receptor-mediated endocytosis enables TCDs to accurately recognize multiple specific pathology-related cells 21, 22. At the organelle level, these dyes may preferentially localize to mitochondria via lipophilic cationic properties, energy-dependent, and transporter-mediated pathways 23, 24, directly regulating cellular energy metabolism and apoptotic pathways 25, 26. We propose this multi-dimensional targeting as a stress-induced selective enrichment (SISE) mechanism, allowing TCDs to serve as precise fluorescent imaging probes and agents for photothermal therapy (PTT), photodynamic therapy (PDT), or the regulation of pathological pathways, thereby constructing a novel “theranostics integration” model 27. Currently, TCDs have achieved significant advances in both fundamental research and application across a range of diseases, including tumor, fibrotic diseases, metabolic disorders, and radiation-induced injuries (RII). For example, IR-34 can also target mitochondrial protein NDUFS1 while completing imaging in cancer cells, significantly inhibiting tumor growth and recurrence 28. Notably, IR-780 identifies glycolytic fibroblasts, and when combined with PTT and PDT, enhances its cytotoxicity towards these cells, providing a potential technique for targeted intervention in fibrotic diseases 27. In addition, TCDs can also exhibit good preventive effects on chronic diseases. For instance, IR-61 can accumulate in the mitochondria of macrophages in adipose tissue, inhibiting macrophage activation and effectively preventing chronic inflammation, weight gain, and metabolic disorders 29. IR-61 can also exert a protective effect on radiation-induced lung injury (RILI). Specifically, it targets the mitochondria of macrophages within irradiated lung tissue, upregulating *Nrf2* and heme oxygenase-1 (HO-1), reducing reactive oxygen species (ROS) and pro-inflammatory factors (IL-1β, IL-6), and alleviating radiation-induced lung tissue damage 30.

**Figure 1:**
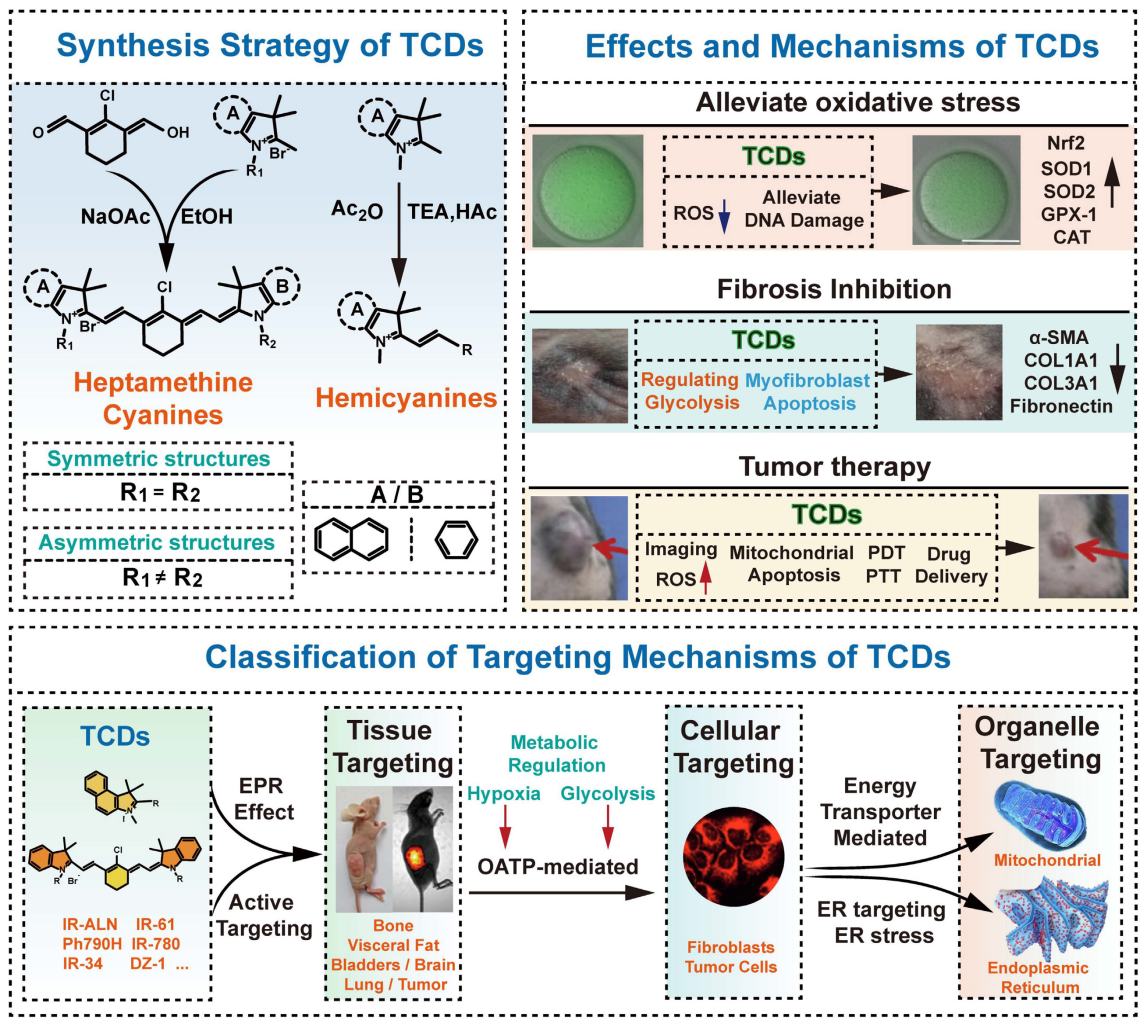
Schematic diagram demonstrate TCD synthesis strategies in this review, alongside an analysis of their hierarchical targeting mechanisms and therapeutic applications for various diseases.

Despite significant progress in TCD-based disease theranostics, several limitations remain: for instance, IR-780 has strong hydrophobicity and may pose potential safety risks due to poor biocompatibility 22; ZWZ-3 only conducted cell/animal efficacy tests, and the *in vivo* metabolic process is still unclear 31; the most important point is that currently most dyes are still in basic research without any clinical trial data, lacking systematic exploration from basic research to clinical applications. Our group has pursued sustained research in this field for many years and has made significant advances in the precise theranostics of various diseases, including tumors, fibrotic diseases, metabolic disorders, and RII. Based on a profound understanding of the field’s current status and development potential, this review (**Figure 1**) systematically summarizes TCDs’ synthesis strategies, targeting mechanism classifications, multi-disease theranostic application progress, and core molecular mechanisms, while proposing key future research directions addressing current challenges. Ultimately, this review aims to provide a comprehensive research perspective, engage more interdisciplinary researchers, and facilitate the advancement of basic research on TCDs and their clinical translation.

## 2. Synthesis Strategies of TCDs

With outstanding optical performance and flexible structural modification space, TCDs have risen to become outstanding NIR fluorescent dyes 32-36. Its excitation wavelength matches the biological NIR optical window, offering outstanding tissue penetration capabilities. Leveraging their wavelength advantages and excellent biocompatibility, heptamethine cyanine dyes and hemicyanine dyes are widely used in bioimaging and phototherapy. The following sections will introduce their synthetic routes and review the latest advancements in functional modifications.

### 2.1 Synthesis of Heptamethine Cyanine Dyes

Heptamethine cyanine dyes (Cy7) are composed of a heptamethine bridge connecting nitrogen-containing heterocyclic chromophores such as indole and benzindole. Their conjugated structure confers NIR optical properties, while the terminal rings, N-substituents, inner positions, and side chains can all undergo structural modifications. Based on molecular symmetry, they can be classified into symmetric and asymmetric types, which exhibit distinct synthetic approaches and functional characteristics 37.

**Figure 2:**
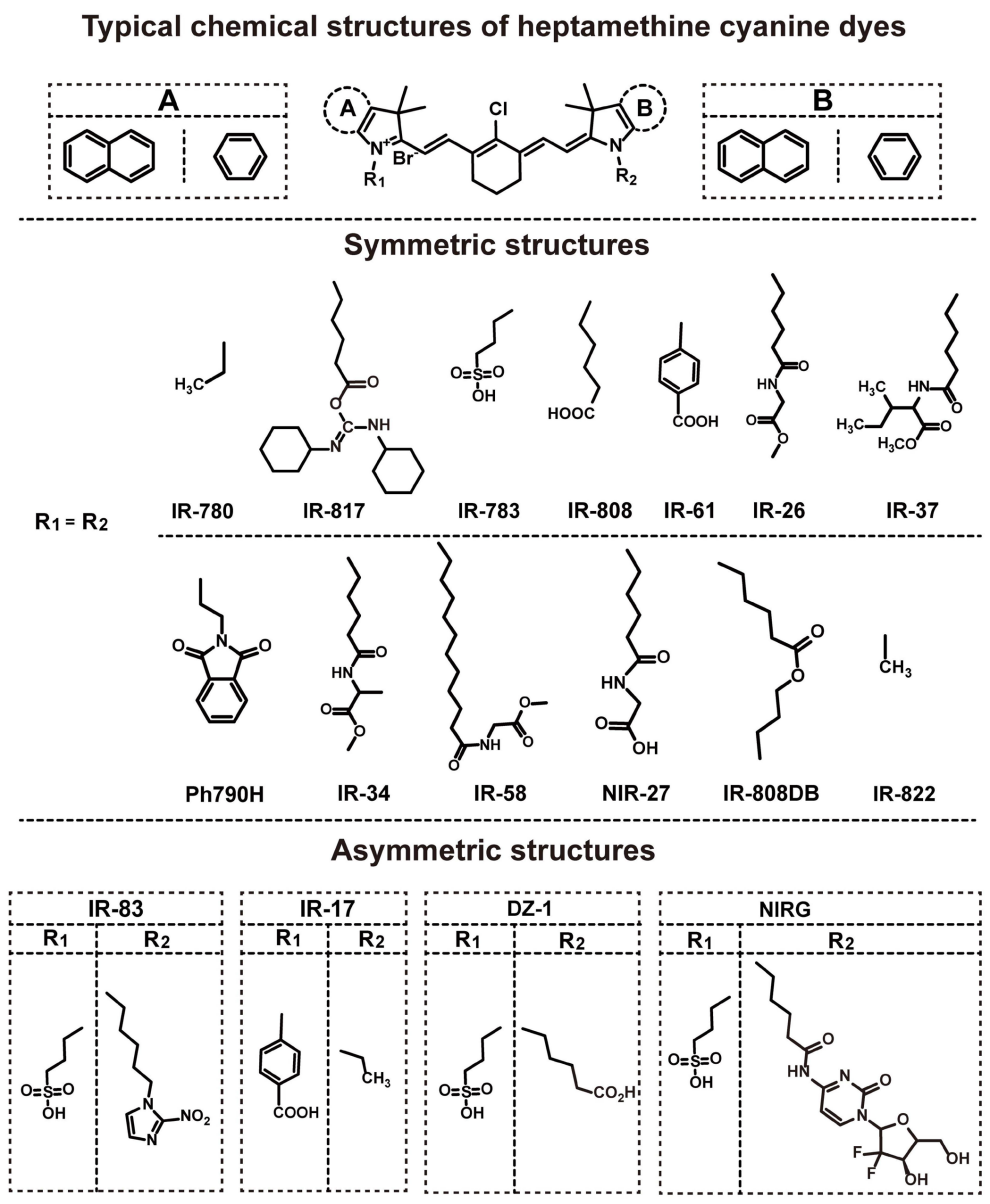
Schematic representation of the typical core structure of heptamethine cyanine dyes and summary of their structural classification. By varying the substituents (R₁ and R₂) and terminal groups, a wide range of derivatives has been developed. These compounds are broadly divided into symmetric (R₁ = R₂) and asymmetric (R₁ ≠ R₂) types, with representative examples shown for each category.

The highly symmetrical planar configuration facilitates π-electron delocalization in symmetric heptamethine cyanine dyes, enabling tunable high-performance NIR absorption and fluorescence in the 700-900 nm wavelength range 38. This type of dye has a high molar extinction coefficient, efficiently capturing light and exhibiting excellent light absorption properties 39. Moreover, strategic structural modifications, notably meso-substitution, serve as a powerful lever to fine-tune their performance 40. Recent work indicates that scaffold tuning can push symmetric heptamethine cyanine dyes into the NIR-II region, supporting their use in deep-tissue imaging and spectral extension 41. Symmetric heptamethine cyanine dyes tend to have balanced electronic structures and predictable photophysical behavior. Asymmetric analogues, however, introduce electronic and steric imbalance through different terminal heterocycles or uneven polymethine substituents, making them easier to tune for solubility, charge distribution, targeting, and stimulus response. Therefore, both symmetric and asymmetric derivatives have been extensively developed as important structural platforms for functional heptamethine cyanine dyes, and representative examples are summarized in **Figure 2**.

**Figure 3:**
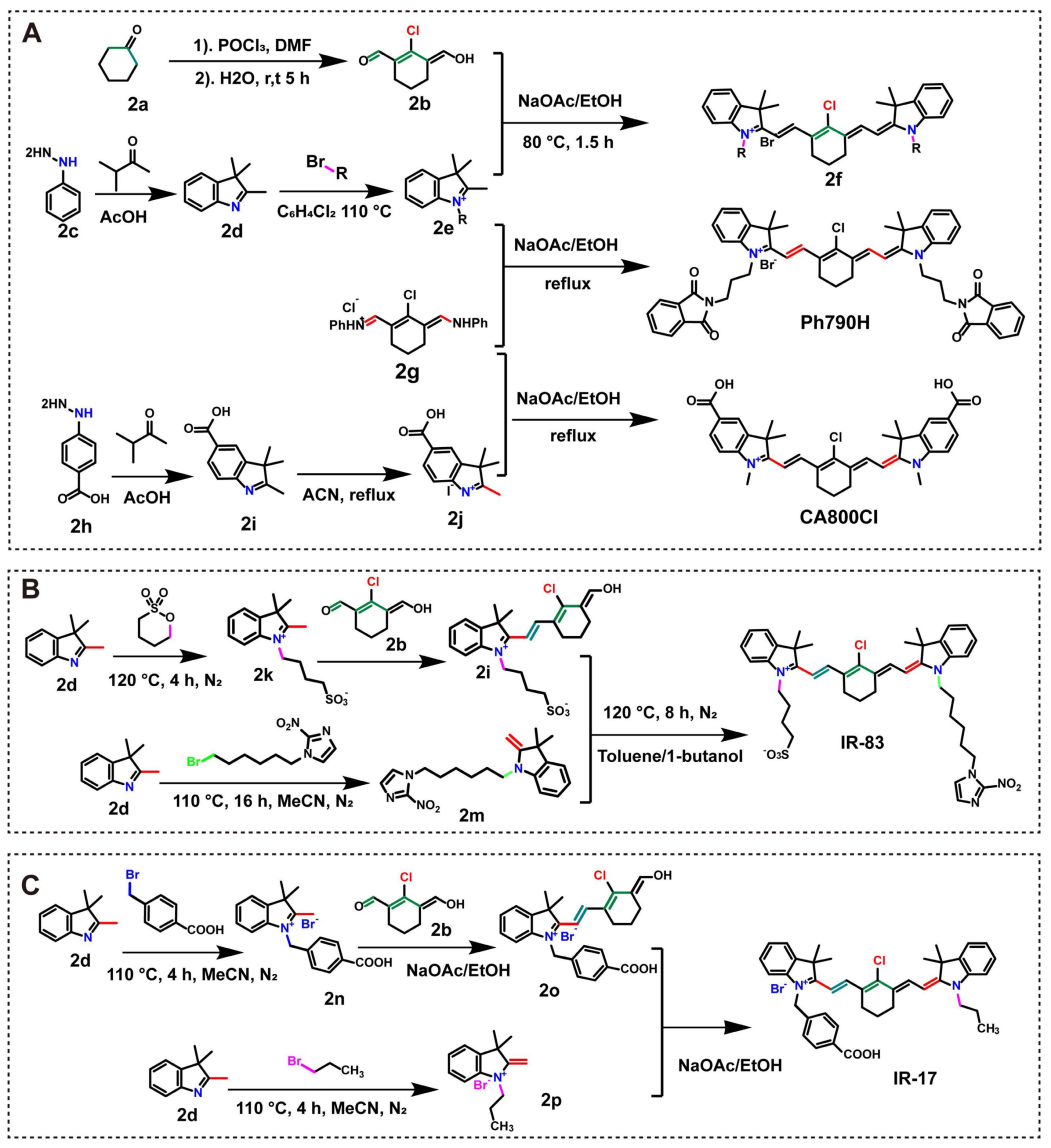
Synthetic routes for heptamethine cyanine dyes. (A) Preparation of symmetric heptamethine cyanine dyes. (B-C) Construction of asymmetric heptamethine cyanine dyes.

Based on these advantages, most of the TCDs in current research are symmetrical heptamethine cyanine dyes, and the synthesis scheme of these dyes can be summarized as follows (**Figure 3A**): Firstly, the synthesis of the meso-chlorocyclohexenecarbaldehyde intermediate (**2b**): Cyclohexanone (**2a**) reacts with a mixture of dimethylformamide (DMF) and phosphorus oxychloride (POCl₃) at room temperature for 5 h, then washed with water and recrystallized from dichloromethane to afford 2-chloro-1-formyl-3-(hydroxymethylene) cyclohex-1-ene (**2b**) 42. Secondly, the synthesis of indole quaternary ammonium salts (**2e**): Phenylhydrazine (**2c**) reacts with 3-methyl-2-butanone under acidic conditions (AcOH) through a one-pot method to generate 2,3,3-trimethyl-3H-indole (**2d**) 43. Subsequently, **2d** reacts with brominated compounds in 1,2-dichlorobenzene at 110 °C for 10 h to yield N-alkylated indole quaternary ammonium salts (**2e**) 44, 45. Thirdly, condensation to form the target dye (**2f**): **2b** and **2e** are refluxed together at 80 °C for several hours in anhydrous ethanol containing anhydrous sodium acetate, affording a series of symmetric heptamethine cyanine dyes (**Figure 2**). The syntheses of Ph790H and CA800Cl differ mainly in the final construction of the cyanine scaffold. For Ph790H, the concluding step employs N-[(3-(benzylidene)-2-chloro-1-cyclohexen-1-yl)methylene]aniline hydrochloride (**2g**) rather than **2b**, reacting **2g** with **2e** in an ethanol/sodium acetate system under reflux for 6 hours to afford the target molecule 46. CA800Cl is prepared through a different route: 4-hydrazidobenzoic acid (**2h**) reacts with 3-methyl-2-butanone in glacial acetic acid to form carboxylated indole (**2i**), followed by heterocyclic salt (**2j**) and final conversion to CA800Cl 47.

Despite the dominance of symmetric heptamethine cyanine dyes, asymmetric analogues are increasingly useful for imaging and therapy because they combine larger Stokes shifts, disease-responsive activation, and improved organelle targeting or tumor retention 48-51. They are typically synthesized through stepwise coupling of two distinct indolium salts. In the synthesis of IR-83 (**Figure 3B**): First, 2,3,3-trimethyl-3H-indole (**2d**) reacts with 1,4-butanesultone at 120 °C for 4 h under a nitrogen atmosphere. This yields 2,3,3-trimethyl-1-(4-sulfobutyl)-3H-indolium (**2k**), which then reacts with **2b** to form **2l**. Second, **2d** reacts with 1-(6-bromohexyl)-2-nitro-1H-imidazole to afford **2m**. Finally, **2l** and **2m** are refluxed at 120 °C for 8 h in a toluene/1-butanol system under nitrogen protection. IR-83 is obtained after purification 52. **Figure 3C** depicts a comparable strategy employed for the synthesis of IR-17. In this case, the key step is the stepwise addition of two distinct functional moieties, leading to the successful assembly of the asymmetric heptamethine cyanine dye 48.

### 2.2 Modification of Heptamethine Cyanine Dyes

In recent studies, the performance of heptamethine cyanine dyes has often been improved through rational structural modification. By coupling different functional groups, researchers can tune their optical properties, enhance their targeting behavior, and introduce therapeutic functions. These strategies mainly include backbone modification, targeting-motif introduction, and therapeutic-agent conjugation.

**Figure 4:**
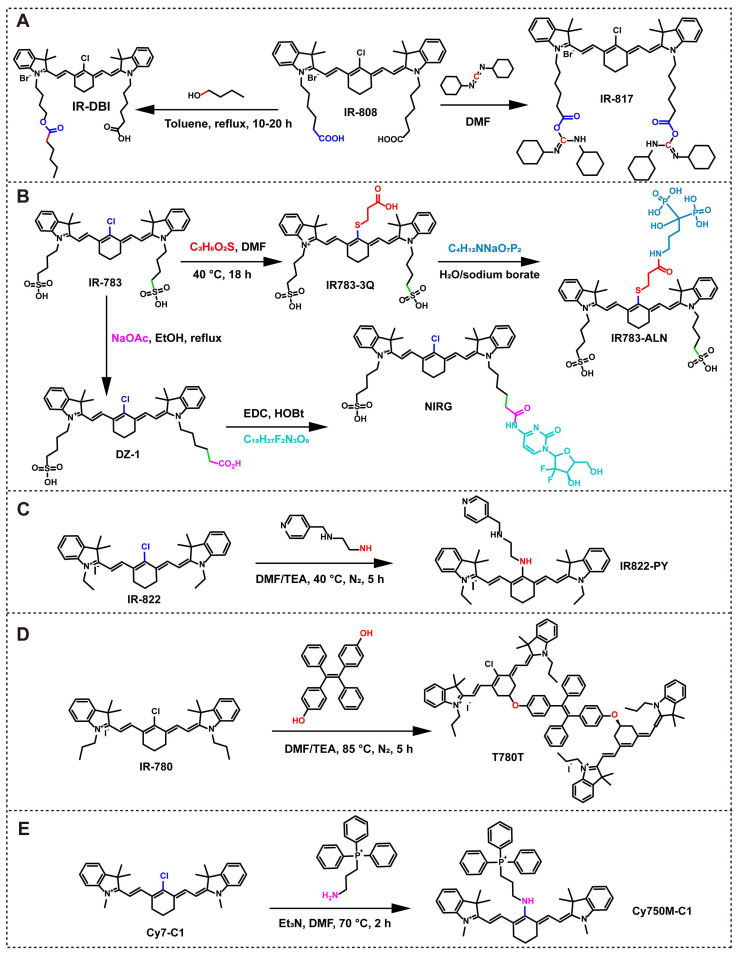
Schematic diagrams of molecular modification for different types of TCDs. (A) Derivatization of IR-808 into IR-DBI and IR-817. (B) Functionalization of IR-783 to yield IR783-ALN and NIRG. (C) Structural modification of IR-822 for the synthesis of IR822-PY. (D) Preparation of T780T based on the IR-780 scaffold. (E) Synthesis of Cy750M-C1 via chemical modification of Cy7-C1.

(1) Coupling of therapeutically active moieties: As shown in **Figure 4A**, Sun* et al.* synthesized IR-817 by reacting IR-808 with dicyclohexylcarbodiimide in DMF at room temperature, followed by imine-bond condensation with carboxyl groups. The resulting dye preferentially localized in cancer-cell mitochondria and selectively inhibited melanoma cells 18. Our group modified the N-alkyl chain of IR-808 and subjected the modified intermediate to reaction in a toluene-n-butanol system for 10-20 h to yield IR-DBI. This compound not only exhibits NIR emission capability but also targets cancer cell mitochondria and selectively suppresses melanoma 53. Its asymmetric amphiphilic structure facilitates cell membrane translocation and mitochondrial targeting, while self-assembles with albumin into complexes to enhance tumor accumulation via the EPR effect 54, 55.

(2) Targeting ligand modification: Based on the significant affinity of bisphosphonates (such as alendronate sodium, ALN) toward hydroxyapatite 16, our research group designed and synthesized a bone-targeted dye IR783-ALN. The specific synthesis route is shown in **Figure 4B.** Firstly, IR-783 was used as a precursor and reacted with 3-mercaptopropionic acid in DMF solvent with stirring at 40 ℃ for 18 h to obtain the intermediate IR-783-3Q; subsequently, the intermediate was placed in a sodium borate buffer system and coupled with ALN to obtain the bone-targeted dye IR783-ALN 56. In addition, in response to the demand for tumor targeting, researchers prepared another derivative of IR-783, DZ-1, using an ethanol/sodium acetate system through reflux technology. Experimental results have shown that DZ-1 not only improves its hydrophilicity, but also has excellent tumor targeting ability. It can be preferentially taken up by hepatocellular carcinoma cells (HCC) and specifically enriched in the subcellular structures of mitochondria and lysosomes 51, 57, 58.

(3) Theranostic integration modification: Jason Boyang Wu *et al*. conjugated DZ-1 with gemcitabine via an EDC-HOBt activation system for 15 h to yield NIRG (**Figure 4B**), a conjugate that markedly enhances the drug’s permeability across the blood-brain barrier (BBB)/blood-tumor barrier (BTB) and prolongs the survival of tumor-bearing mice 59. Similarly, Meng *et al*. fabricated the smart probe IR822-PY (**Figure 4C**) by stirring the pH-responsive receptor PY with IR-822 in triethylamine at 40 °C for 5 h under nitrogen. This probe integrates dual functions of pH-sensitive imaging and PTT 60. In another approach, Zhao *et al*. reacted IR-780 with tetraphenylethylene (TPE) in a triethylamine-DMF system at 85 °C for 4 h to yield T780T (**Figure 4D**). The twisted conformation of TPE elevates the photothermal conversion efficiency (PCE) to 38.5% through intramolecular motion in the excited state 61. Furthermore, Shen *et al*. introduced triphenylphosphine (a mitochondrial targeting moiety) at the meso-position of heptamethine cyanine dyes 62-64, with the reaction proceeding in a triethylamine/DMF system at 70 °C for 2 h to afford Cy750M-C1 (**Figure 4E**). Building on this, subsequent co-assembly with DSPE-PEG₂₀₀₀ and DSPE-PEG₂₀₀₀-FA yielded Cy750M-C1-FA-NPs, which achieved remarkable improvements in water solubility, tumor targeting specificity, and intratumoral accumulation 65.

Compared with the above cases, stimuli-responsive activation provides an important pathway for the intelligent design of TCDs 66. A typical example is the pH-responsive amino heptamethine cyanine dyes probe I_2_-IR783-Mpip, in which acid triggered fluorescence recovery is achieved by modifying the median position with N-methylpiperazine, and singlet oxygen generation is enhanced by protonated PET inhibition. The probe exhibits a wide NIR absorption range of 820-950 nm under acidic conditions and can mediate PDT under irradiation at approximately 850 nm 67. In addition, higher specificity can be achieved through a dual locking strategy, such as the semi cyanine-based nanoprobe DL-P, which can simultaneously respond to lysosomal acidic pH and protease B, thereby releasing CyNH_2_, activating NIR fluorescence, and activating therapeutic functions 68. Enzyme activated cyanine probes also provide useful examples for intelligent TCD design. For example, QcyP is a switch type NIR probe based on heptamethine cyanine, which uses phosphate monoesters as alkaline phosphatase (ALP) response units. ALP mediated dephosphorylation triggers rearrangement of the conjugated π-electron system and activates NIR fluorescence, enabling imaging of endogenous ALP in cells and HepG2 tumor-bearing mice 69. With pH/enzyme-responsive or dual-triggered designs, TCD systems can convert disease-microenvironment cues into linked imaging-therapy outputs, improving specificity while reducing off-target effects.

### 2.3 Synthesis of Hemicyanine Dyes

**Figure 5:**
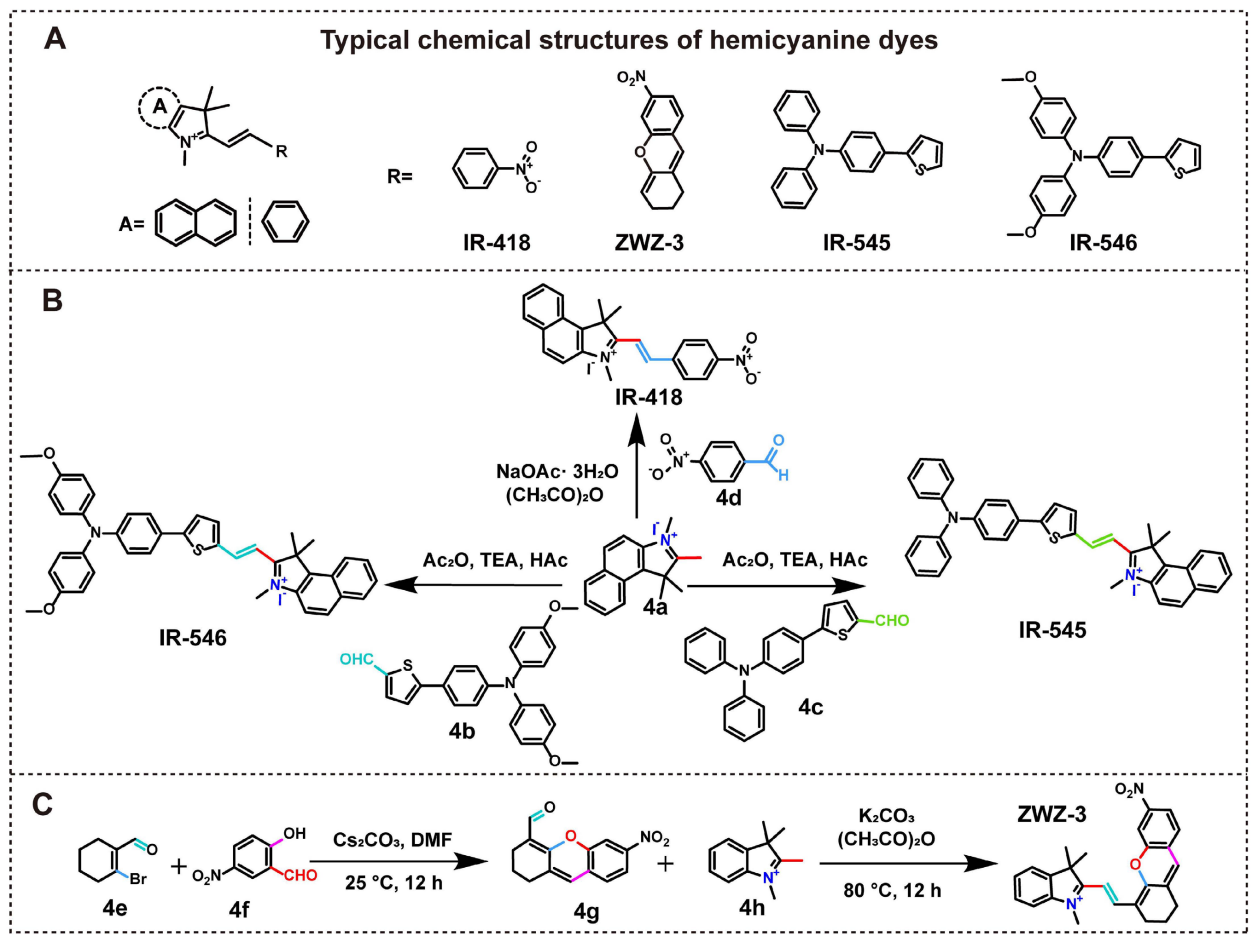
Typical chemical structures and synthetic pathways of representative hemicyanine dyes. (A) Typical chemical structures of hemicyanine dyes. (B) Synthetic pathways leading to IR-418, IR-545, and IR-546 from the precursor 4a. (C) The synthetic route of ZWZ-3 from its starting precursors.

The unique donor-acceptor (D-A) molecular structure, considerable Stokes shift, and excellent biocompatibility make the hemicyanine dyes a commonly used fluorescent probe for biomedical imaging 70-72. Its typical structure is the D-π-A configuration (**Figure 5A**), where the conjugated π system bridges the electron donor and the nitrogen-containing heterocyclic acceptor. Thanks to the flexible molecular design, changing the donor unit, acceptor unit, or polyacetylene chain will lead to significant changes in the dye’s performance. This type of material synthesis process is simple and straightforward. The core of the process is the condensation reaction between aromatic aldehydes and nitrogen-containing heterocycles to form a conjugated structure. The following text will introduce relevant synthesis examples.

The experiment synthesized IR-546 using 1,2,3,3-tetramethyl-3H-indolium iodide (**4a**) and 5-(4-(bis(4-methoxyphenyl)amino)phenyl)thiophene-2-carbaldehyde (**4b**) as raw materials. The two reactants were first fully dissolved in acetic anhydride, and then triethylamine and acetic acid were added. The reaction was carried out at 60 ℃ with stirring for 1 hour. This route achieved ideal reaction results and the yield of the product was considerable 73. The synthesis steps of IR-545 are the same as those of IR-546, except that **4b** is replaced by 5-(4-(diphenylamino)phenyl)thiophene-2-carbaldehyde (**4c**) 74. IR-418 was synthesized using **4a** and 4-nitrobenzaldehyde (**4d**) as raw materials. The reaction was carried out at room temperature in a mixture of sodium acetate trihydrate and acetic anhydride for 1 day. The product is shown in **Figure 5B** 75. The preparation process of ZWZ-3 differs from the aforementioned single condensation reaction (**Figure 5C**).

Firstly, 2-bromocyclohex-1-ene-1-carbaldehyde (**4e**) and 2-hydroxy-5-nitrobenzaldehyde (**4f**) were fully dissolved in the cesium carbonate modified DMF solvent and stirred at room temperature for 12 hours to complete the preparation of intermediate 6-nitro-2,3-dihydro-1H-xanthene-4-carbaldehyde (**4g**). Subsequently, using **4g** and **4h** as reaction raw materials, relying on the composite system of potassium carbonate and acetic anhydride, a constant temperature reaction was carried out at 80 °C for 12 hours, and finally afforded the target dye ZWZ-3 31.

In summary, the synthetic system of TCDs centers on structural tunability and is guided by functional adaptability, creating a multi-level framework for preparation and modification. By employing symmetrical or asymmetrical molecular synthesis design, researchers can flexibly control the NIR optical properties of heptamethine cyanine dyes. The core regulatory mechanism lies in precisely controlling the π-electron delocalization state and rationally arranging the functional groups on the molecular surface, thereby achieving the targeted optimization of the dye’s performance. Researchers are increasingly moving past traditional synthetic routes by employing cyanine dyes as luminescent centers to build structurally defined dendritic nanodots. By progressively assembling polylysine dendritic structures with Cy3/5/7 as the core, fluorescent-tunable, photostable, and biocompatible nanodots can be prepared 76. The subsequent conjugation of targeting or therapeutic groups demonstrates the advantage of integrated diagnosis and treatment, effectively promoting the clinical translation of TCDs.

### 2.4 Structure-Activity Relationships of TCDs

Based on clear structure-activity relationships (SARs), TCDs can effectively modulate their optical properties, physicochemical characteristics, and targeting capabilities with only minor structural adjustments. The heptamethine polymethine skeleton is the core of TCDs. Its conjugated methyl unit dominates the basic optical properties of NIR 20, 28, 53, 77. The extended π system ensures stable NIR fluorescence and enables precise control of the amphiphilic balance. This strategy achieves two benefits in one: it enhances the tissue permeability of short-chain dyes and alleviates the common toxicity and aggregation quenching problems of long-chain alkyl substituents 42,53. With its lipophilic cationic properties and OATP transport mechanism, this scaffold can penetrate deep into tissues and target accumulation at high metabolic lesions. Further locking the cyclohexene ring in the poly(methyleneimine) bridge to increase structural rigidity not only reduces non-radiative decay but also significantly enhances its selective retention effect at the tumor site 28, 53, 78.

The key to determining the solubility, pharmacokinetics, and subcellular distribution of TCDs lies in peripheral functional modifications rather than the core skeleton. For example, the carboxyl-substituted benzyl-N-alkyl chain of IR-61/817 confers moderate water solubility and achieves mitochondrial targeting through membrane potential driven mechanisms 20, 29. Sulfonic functionalized derivatives such as DZ-1 and IR-783 exhibit different advantages: they can improve water solubility, accelerate clearance *in vivo*, and do not weaken the original tumor binding ability 78. Amino acid modification (such as NIR-27 grafted with glycine) can effectively improve biocompatibility 79. Asymmetric amphiphilic substituents such as IR-DBI enhance the binding affinity of albumin, thereby optimizing pharmacokinetics and promoting tumor targeted delivery through the EPR effect 53.

Based on the advantages of this structure, the derivatization strategy can endow TCDs with new functional characteristics while retaining their inherent targeting properties. Representative approaches include drug conjugation (e.g., 780-5FU and FTS-148), twisted structural design to suppress π-π stacking (e.g., T780T), and carrier-assisted formulations such as BSA@IR-817 and HSA@IR-DBI 20, 53, 78, 80. The use of this strategy can significantly improve delivery efficiency and effectively suppress fluorescence quenching caused by aggregation. This not only improves the final effect of image-guided therapy, but also perfectly preserves the targeting specificity of the system. The SARs of TCDs are a multidimensional system. The heptamethine skeleton determines the basic optical and targeting properties; side chain modification dominates the physicochemical and biological manifestations; and derivatization further enhances the clinical application value. Based on this structure function association, we were able to deduce the precise targeting mechanism of TCDs, which laid the foundation for the discussion of various treatment strategies in the following text.

## 3. Classification of Targeting Mechanisms of TCDs

### 3.1 Tissue Targeting Specificity of TCDs

The prerequisite for achieving precise diagnosis and treatment of TCDs is to overcome the challenge of targeting specificity. By utilizing passive or active targeting mechanisms to promote efficient enrichment of dyes in the lesion area, it can lay the foundation for their subsequent applications.

#### 3.1.1 Passive Targeted Accumulation of TCDs Relying on the EPR effect

**Figure 6:**
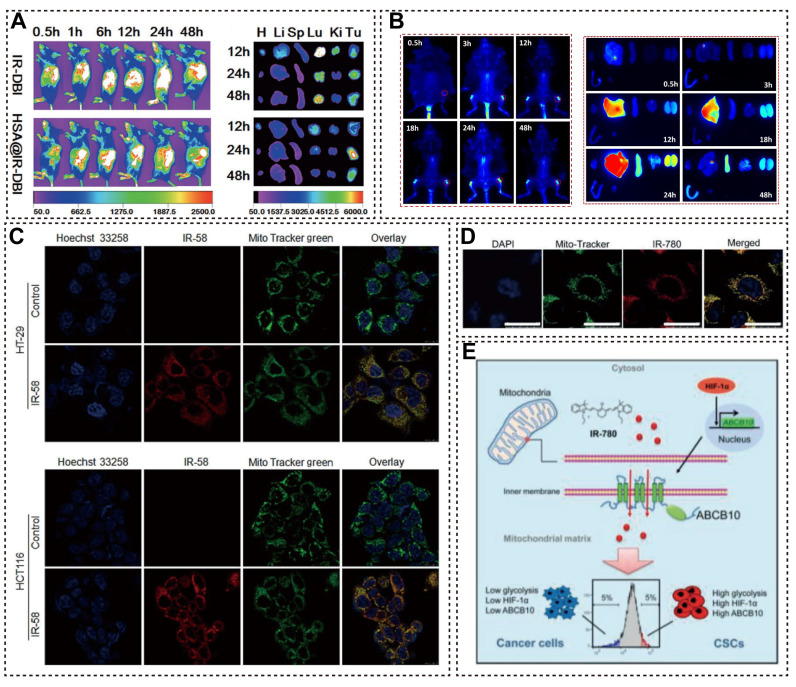
(A) Representative *in vivo* and *in vitro* images of tumor-bearing mice following administration of HSA@IR-DBI and free IR-DBI. Adapted with permission from 53. Copyright 2017, John Wiley and Sons. (B) NIR fluorescence images of bone tissue and major organs dissected from mice taken at different time points after post-injection of IR-ALN. Adapted with permission from 56. Copyright 2024, John Wiley and Sons. (C) The co-localization images of IR-58 and mitochondrial-specific tracer in different cells. Adapted with permission from 101. Copyright 2018, BMJ Publishing Group Ltd. (D) Schematic illustration of HIF-1α/glycolysis-dependent regulation of mitochondrial transporter ABCB10 in characterizing CSCs using IR-780. Adapted with permission from 114. Copyright 2018, John Wiley and Sons.

Passive targeting utilizes the physiological differences between lesions (such as tumors or inflamed tissues) and healthy tissues to drive dye accumulation 19. Central to this strategy is the EPR effect. This strategy is based on the EPR effect: the endothelial gap of diseased blood vessels widens (about 100-600 nm) and lymphatic reflux is obstructed, making it easy for molecules or nanoparticles of appropriate size to penetrate the interstitium and remain 81, 82. In the TCDs field, this property is utilized to design dyes that bind to serum albumin (BSA/HSA) to construct nanocomplexes 83, 84. Albumin, as a dual functional carrier, can enhance photostability and inhibit aggregation quenching, while actively promoting tumor enrichment based on the EPR effect 85-87. Wang *et al*. constructed BSA@IR-817 nanoparticles, enhancing the binding through both covalent and supramolecular interactions. This complex exhibited superior tumor targeting properties compared to the free IR-817, and the fluorescence peaked 6 hours after injection and remained stable for 24 hours 20. Zhao *et al.* developed T780T nanoparticles, which self-assembled into approximately 200 nm aggregates in water. Utilizing the EPR effect, it achieved sustained tumor enrichment for 96 hours 61. Furthermore, Tan *et al*. revealed that IR-DBI could bind tightly to site II of HSA and self-assemble into the drug-protein complex HSA@IR-DBI in plasma. Benefiting from the EPR effect, HSA@IR-DBI exhibited higher preferential tumor accumulation in tumor tissues relative to free IR-DBI. Even at 24 h, high-contrast signals between tumors and adjacent marginal regions remained observable (**Figure 6A**) 53. Additionally, studies have shown that the rigidity of nanoparticles has recently become an important physicochemical parameter for regulating tissue permeability and accumulation. For example, mechanically adjustable dye core polylysine dendrimers exhibit a biological trade-off related to rigidity: Harder nanodots promote deep penetration in 3D tumor spheres, while softer nanodots exhibit longer blood circulation time and enhanced tumor accumulation 88. The preceding scenario demonstrates that the creation of complexes between TCDs and proteins properly fits the size range of the EPR effect, which can considerably boost the retention effectiveness and longevity of TCDs in tissues. This passive targeting technique effectively addresses the issue of low retention efficiency of free TCDs in tissues. Although the EPR effect has been widely validated in preclinical animal models, its actual efficacy in human tumor treatment remains controversial. The high complexity of human tumors constitutes the main bottleneck. The dense microenvironment, high interstitial hydraulic pressure, and abnormal vascular permeability form multiple physiological barriers. As a result, the *in vivo* accumulation of TCD nanomedicine is extremely unstable, making it difficult to predict whether it is in different tumor types or within a single lesion. This variability greatly reduces the clinical reliability of passive targeted therapy 89. The inherent heterogeneity of tumors can lead to an uneven spatial distribution of EPR-driven TCDs 90. Especially in cases where the EPR effect is weak or unstable, a single passive mechanism cannot fully unleash the diagnostic and therapeutic potential of TCDs. Therefore, active targeting must be introduced as a core supplement. This technology not only promotes cellular endocytosis, but also enhances tissue specificity, thereby significantly improving overall therapeutic efficacy.

#### 3.1.2 Active Targeting Mediated by Molecular Modifications

Compared to passively waiting for microenvironmental conditions, active targeting has adopted more proactive intervention measures. Researchers can deliver drugs directly to damaged tissues by adjusting their physicochemical properties or adding targeting groups. This approach eliminates excessive dependence on the EPR effect and ensures delivery reliability even in cases of weak vascular leakage or high tumor heterogeneity. From a molecular mechanism perspective, charge, size, and hydrophobicity determine the permeability and retention ability of dyes 91, 92; especially lipophilicity plays a central role in promoting dye penetration into adipose tissue 93. Consider IR-61: because it is both lipophilic and cationic, an intraperitoneal injection allows it to bypass rapid clearance and build up steadily in visceral adipose tissue, aided by the naturally sluggish blood flow in that region 94. The important approach to achieving active targeting lies in introducing functional groups capable of high-affinity binding to specific tissues into dye molecules via chemical modification. Our group developed IR783-ALN, which mitigates nonspecific protein binding *in vivo* via chlorine-atom substitution on the Cy7 scaffold, while the incorporated bisphosphonate moiety confers targeted bone tissue recognition. After intravenous injection, its fluorescence signal in the distal femur reaches its peak at 24 h and lasts for up to 48 h, indicating strong bone targeting specificity (**Figure 6B**) 56. This strategy supplemented the poor targeting of free TCDs, and the alteration of particular molecules improves TCDs targeting.

### 3.2 Cellular Targeting Specificity of TCDs

OATPs are part of the solute carrier organic anion polypeptide transporter family (*SLCO*), which includes six subfamilies (OATP1-OATP6). These transporters can transport many different natural and foreign molecules into cells, providing a basis for TCDs to target specific cells 21.

#### 3.2.1 OATPs-Mediated Targeted Translocation of TCDs into Fibroblasts

Studies have shown that the specific uptake of TCDs by fibroblasts and their activated subtypes (such as cancer-associated fibroblasts, CAFs) is mainly mediated by specific OATP subtypes, and this process is closely related to cellular glycolytic metabolism 95. Many research cases have also confirmed this viewpoint. For instance, Meng *et al*. demonstrated that IR-780 can specifically target activated, highly glycolytic fibroblasts via *SLCO2A1*-mediated transmembrane transport, and this preferential accumulation phenotype is regulated by glycolytic metabolism 96. Wang *et al*. further confirmed that the extensive accumulation of IR-780 in CAFs is associated with high expression of OATP. After intervention with BSP (a competitive OATP inhibitor) and *SLCO2A1* small interfering RNA (siRNA), the uptake of IR-780 by CAFs was significantly reduced, indicating that OATPs (particularly OATP2A1) are the core mediators of specific IR-780 uptake in CAFs 97. A recent study by Wu *et al*. extended these findings by finding that the entry of IR-780 into fibroblasts depends on the high expression of the OATP1B2 subtype 22. Chen *et al*. demonstrated that highly active glycolysis and *SLCO2A1* synergistically drive IR-780 uptake in fibroblasts. In fibroblasts cultured under 5% hypoxia, the expression levels of hypoxia-inducible factor-1α (HIF-1α), glycolytic enzymes (LDHA, LDHB, LDHC), and *SLCO2A1* were significantly upregulated; this effect was reversed by LW6 (a HIF-1α inhibitor), indicating that HIF-1α is a master regulator of the glycolytic phenotype in fibroblasts and *SLCO2A1*-mediated IR-780 uptake 27. Wang *et al*. discovered that glycolysis enhanced the intracellular retention of IR-780. After using* SLCO4A1* siRNA, the uptake of IR-780 by fibroblasts and myofibroblasts was significantly inhibited 98. The mechanism of the inhibitory effect of α-KG on HIF-1α is as follows: the HIF-1α/*SLCO4A1* pathway regulates dye internalization, while the active accumulation of the dye is promoted by the *SLCO2A1* transporter through glycolysis activity 99.

#### 3.2.2 OATPs-Mediated Targeted Translocation of TCDs into Tumor Cells

In addition to fibroblasts, OATPs are also a key entry point for various tumor cells to uptake TCD and are closely related to HIF-1α mediated hypoxia signals 100. Blocking OATPs by BSP can significantly reduce the accumulation of IR-817 in melanoma cells 18, which is also involved in the uptake of IR-58 in HT-29 and HCT116 cells 101. Further research has shown that the entry of IR-780 into cells depends on energy metabolism, plasma membrane potential, and *OATP1B3*, rather than endocytosis or ABC transporters. It can also avoid the efflux of *ABCB1*/*ABCC1*, thereby maintaining intracellular retention 102. Hypoxia is closely related to OATP expression. Wu *et al.* found that in prostate cancer, hypoxia can promote HIF-1α binding to the *OATP1B3* promoter and upregulate its transcription, thereby enhancing MHI-148 uptake. Even when *OATP1B3* is knocked down under normoxic conditions, dye uptake still decreases by about 30%, indicating that this process is jointly regulated by hypoxia signaling and OATP expression 99. The canine model further indicates that HIF-1α stabilization can promote *OATP1B3*/*2B1* expression, while rifampicin and other OATP inhibitors can weaken this effect 103. Interestingly, OATP activation can also promote drug crossing over the BBB and BTB, and is not entirely dependent on strict hypoxia 59, 104. Qin *et al*. also confirmed that HIF-1α can bind to the *OATP1B3* promoter HRE sequence to drive transcription, while Abi-DZ-1 enters tumor cells through the HIF-1α/OATP axis 78. Overall, the tumor targeting of TCDs mainly comes from OATP mediated transport and is jointly influenced by HIF-1α hypoxia signaling and glycolytic metabolism. A profound understanding of this mechanism can provide a clear mechanistic basis for the development of highly specific dye probes. It is worth noting that the cellular targeting of TCDs is not limited to OATP-mediated uptake of traditional small molecule dyes. Recently, Cy5 based supramolecular amino acid encoding nanodots have further demonstrated that the cyanine dye skeleton can also expand its cellular delivery function through nanoplatform design. This type of cyanine derived nanoplatform utilizes surface amino acid motifs to regulate protein recruitment, serum stability, and pH/ion responsive phase separation processes, achieving efficient and serum resistant cytoplasmic protein delivery 105. It provides a new design approach for the functional expansion of TCDs in targeted delivery at the cellular level.

### 3.3 Organelle Targeting Specificity of TCDs

We summarized and analyzed the subcellular localization (as shown in **Table 1**), and the results showed that the vast majority of TCDs exhibited specific localization to mitochondria. Mitochondria are the core of cellular energy metabolism and the hub of cell apoptosis regulation, and their functional abnormalities are closely related to the occurrence and development of various diseases 106. Therefore, developing mitochondrial-targeted compounds is a highly promising intervention strategy 107, 108. Numerous studies have confirmed that mitochondrial targeting ability is a core functional attribute of TCDs 109. Some studies have indicated that the mitochondrial membrane potential of tumor cells is higher than that of normal cells, which is the basis for the selective enrichment of the lipophilic cationic compound rhodamine 123 110-113. The mitochondrial targeting of TCD derivatives may not depend on the mitochondrial membrane potential, but rather achieved via energy-dependent or transporter-mediated pathways, thereby exhibiting diverse targeting mechanisms. Chen *et al*. confirmed that IR-780 can target mitochondria through stress induction. The preventive application of IR-780 can significantly inhibit the process of cell apoptosis triggered by ischemia and oxidative stress, thereby effectively maintaining cardiac function and delaying heart failure. Further mechanism analysis indicates that IR-780 induces a rapid decrease in mitochondrial membrane potential by specifically binding to F_0_F_1_-ATP synthase in the mitochondrial respiratory chain, thereby inhibiting energy metabolism and placing mitochondria in a “relative resting state”. In addition, the compound can effectively inhibit the abnormal opening of the mitochondrial permeability transition pore (mPTP) by blocking mitochondrial calcium overload, exerting a cardioprotective effect 23.

Zhang *et al*. found that IR-780 can selectively localize to mitochondria after internalization, but its uptake mode is different from typical lipophilic cations. Even if 10 mM carbonyl cyanide-4-trifluoromethoxyphenylhydrazone (FCCP) induces mitochondrial depolarization, the internalization of IR-780 is still largely unaffected, indicating that mitochondrial membrane potential is not the main driving force for its accumulation 102. In contrast, cyanine dyes such as IR-58 exhibit stronger mitochondrial selectivity. Huang *et al.* confirmed through Mito-Tracker Green co localization that IR-58 can target the mitochondria of HT-29 and HCT116 colorectal cancer cells (**Figure 6C**). Low temperature treatment and glycolysis or oxidative phosphorylation inhibitors significantly reduced its uptake, indicating that the mitochondrial enrichment of IR-58 depends on cellular metabolic energy supply 101. Wang *et al.* reported that IR-780 can be enriched in mitochondria, and its uptake is closely related to the regulation of HIF-1α in cancer stem cells (CSCs). Upregulation of HIF-1α can enhance the activity of the mitochondrial transporter *ABCB10*, thereby promoting the specific uptake of IR-780 (**Figure 6D**) 114. Besides mitochondria, TCDs can also be used for endoplasmic reticulum (ER) targeting. ER-Cy-poNO₂ can preferentially accumulate in the ER of tumor cells and enhance the anti-tumor effect of PDT by inducing strong ER stress and immunogenic cell death (ICD) 115. Chen *et al*. further encapsulated ER specific dyes in Ds-sP/TCPP-TER nanoparticles, which targeted ER under NIR irradiation and amplified ICD response by intensifying ER stress 116. Some TCDs are not limited to a single organelle and have dual targeting capabilities. Ph790H can simultaneously locate in mitochondria and ER, and induce tumor cell death through mitochondrial damage, ROS generation, and ER stress 46; Abi-DZ-1 targets mitochondria and lysosomes, promoting apoptosis and inhibiting tumor proliferation by disrupting mitochondrial function 78. Therefore, the subcellular localization of TCDs not only supports their imaging and therapeutic effects, but also provides a basis for observing organelle related lesions and designing targeted therapies.

As shown in **Table 1**, the targeting characteristics of over 30 TCDs indicate that these dyes have practical value in the treatment of cancer, fibrosis, and metabolic diseases. Its mode of action can be summarized as “tissue-cell-subcellular”: it is enriched in tumor or bone tissue at the tissue level, recognizes specific cell types such as fibroblasts at the cellular level, and is mainly localized in mitochondria (>90%) at the subcellular level. Some dyes also have dual targeting capabilities for mitochondria/lysosomes. This mitochondrial bias not only reflects its structural design characteristics, but also highlights the importance of mitochondrial regulation in treatment. **Table 1** further indicates that chemical structure fine-tuning can significantly affect the biological effects of TCDs. The skeleton of heptamethine mainly determines its color and basic targeting characteristics, while the side chain structure directly affects its water solubility, clearance rate, tumor retention, and mitochondrial affinity. Derivatives bearing sulfonic acid moieties, such as DZ-1 and IR-783 can improve water solubility and promote *in vivo* clearance. Derivatives functionalized with amphiphilic moieties, such as IR-DBI and IR-61 can help enhance tumor retention and mitochondrial binding. There are also differences in the distribution of different dyes in the body: IR-26 and IR-34 can maintain longer blood circulation, while most dyes have a clearance time of less than 48 hours in major organs such as the liver and kidneys. IR-783 shows good potential for guiding treatment due to its long-term retention in tumor tissues. Overall, these dyes have translational value in terms of safety and pharmacokinetics, but their long-term toxicity and specific metabolic pathways still need further clarification.

Overall, the differences between TCDs and squarylium/merocyanine dyes are not only in their NIR spectral performance, but also in their small molecular frameworks that can integrate multiple functions such as lesion enrichment, transporter mediated internalization, organelle localization, and therapeutic regulation 117. Squarylium cyanine is highly attractive for stable NIR imaging and sensing, while merocyanine dyes exhibit excellent optical tunability and potential for NIR-II phototherapy 118. However, their disease targeting often relies more on external functional modifications, nanocarrier construction, or responsive probe design 119. In contrast, TCDs exhibit more significant structural associations and pathological targeting features. At the tissue level, selective accumulation can be achieved through passive EPR mediated retention and active ligand targeting. At the cellular level, the OATP-mediated transmembrane transport mechanism, which is closely related to hypoxia signaling and glycolysis reprogramming can achieve disease-related cell recognition. At the subcellular level, the specific localization of mitochondria (with minor distribution in lysosomes or ER) ensures the functional layout of therapeutic regulation. These collaborative mechanisms construct a hierarchical targeting mode of tissue/cell/organelle, enabling TCD design to accurately match the pathological microenvironment. Importantly, this targeted behavior is directly related to the synthesis and modification strategies described in chapter 2, which determine the targeting sequence, binding affinity, and stimulus response characteristics of TCDs. Therefore, the structure function synergy of TCDs provides a unique strategy to overcome the limited specificity of traditional diagnosis and treatment platforms, making them particularly suitable for hierarchical precision diagnosis and treatment.

## 4. Therapeutic Effects and Molecular Mechanisms of TCDs

### 4.1 Antioxidant and Cytoprotective Effects Mediated by the Nrf2 Pathway

As is well known, oxidative stress is a key driving factor for cell damage. Exposure to ionizing radiation (IR) or hydrogen peroxide (H₂O₂) can trigger the accumulation of large amounts of ROS within cells, resulting in DNA double-strand breaks (DSBs), apoptosis, tissue damage, and impaired self-healing ability 120, 121. Given their intrinsic mitochondrial-targeting ability, TCDs can directly modulate mitochondrial oxidative stress and protect cellular function. This property has contributed to their therapeutic effects in several oxidative stress-related disease models 122, as shown in the following specific cases.

**Figure 7:**
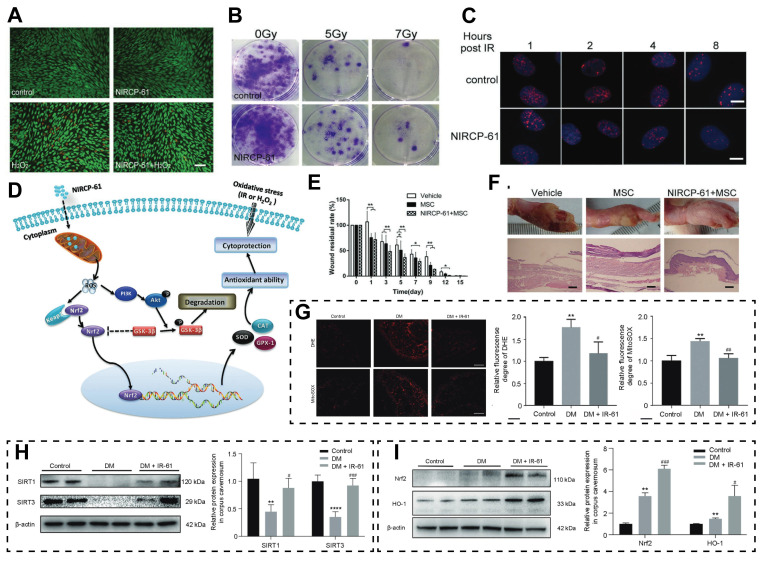
(A) The results of dual staining with calcein-AM and PI under different treatment conditions. (B) Images of hUCMSCs colony formation. (C) Images of γ-H2AX in the nuclei of hUCMSCs after IR. (D) Mechanisms of NIRCP-61 against oxidative stress. (E-F) The healing status of wounds at different time points. Adapted with permission from 123. Copyright 2016, John Wiley and Sons. (G) DHE and MitoSOX staining and their quantitative results. (H) Representative images and quantitative results of Western blot analysis for SIRT1 and SIRT3. (I) Western blot analysis and quantification results of Nrf2 and HO-1 in tissues. Adapted with permission from 126. Copyright 2025, Wolters Kluwer Medknow Publications.

NIRCP-61 has shown strong cell protective potential in preclinical studies. In the oxidative stress models induced by H_2_O_2_ and IR, NIRCP-61 can effectively alleviate damage to mesenchymal stem cells (MSCs). The results of the calcein AM/PI co-staining and colony formation experiments (**Figure 7A-B**) showed that NIRCP-61 pretreatment significantly reduced stress-induced cell death. The mechanism research results indicate that NIRCP-61 provides dual protection by reducing intracellular total ROS levels and decreasing IR-induced DNA damage, which is confirmed by the significant decrease in γ-H2AX expression (**Figure 7C**) 123. This process can accelerate the dissociation of Nrf2 from its inhibitory partner protein Keap1, promote Nrf2 nuclear translocation, and thereby activate the transcriptional activity of downstream antioxidant response elements (AREs). At the same time, the increase in intracellular ROS levels can trigger activation of the PI3K/Akt pathway, which can further amplify antioxidant responses by promoting phosphorylation of Nrf2, Akt, and their negative regulatory factor GSK-3β 123, 124. As shown in **Figure 7D**, Nrf2 activation upregulated antioxidant enzymes such as SOD1, SOD2, glutathione peroxidase-1 (GPX-1), and CAT, indicating that NIRCP-61 can enhance cellular antioxidant defense. Further animal experiments have shown that this protective effect is not limited to *in vitro* systems, but has also been validated in various *in vivo* models. For example, in a rat model of myocardial infarction, transplantation of human umbilical cord mesenchymal stem cells (hUCMSCs) pretreated with NIRCP-61 can significantly reduce the left ventricular infarct area. In addition, in the combined model of radiation injury and trauma injury, NIRCP-61 pretreatment accelerated wound healing on days 3 and 5 after injury (**Figure 7E**). Similarly, in a hindlimb injury model induced by high-dose radiation (40 Gy), NIRCP-61-pretreated hUCMSCs effectively improved skin ulcers and edema, and promoted dermal tissue regeneration (**Figure 7F**). Wang *et al*. further elucidated the potential molecular mechanism of IR-61 and pointed out that IR-61 can induce transient release of superoxide anions in mitochondria and cytoplasm 26. Specifically, IR-61 preferentially enters adipose tissue macrophages (ATMs) and localizes to mitochondria. Via the ROS/Akt/Acly pathway, mitochondrial complexes and OXPHOS activity are enhanced, inhibiting M1 inflammatory activation, and improving obesity, insulin resistance, and fatty liver. This case demonstrates that TCDs can serve as immunometabolic modulators.

IR-61 exhibits a similar protective effect in the diabetic bladder dysfunction (DBD) model. Wang *et al*. found that IR-61 can alleviate mitochondria-associated apoptosis in the bladder tissues of DBD rats by inhibiting cellular and mitochondrial superoxide (MitoSOX) levels. Specifically, IR-61 treatment can significantly reverse downregulation of Nrf2, SOD-1, SOD-2, and HO-1 expression, while inhibiting Keap-1 upregulation in the bladder tissues of the DBD + IR-61 group, further confirming that the protective effects of IR-61 is closely related to the activation of the Nrf2 pathway 125. A study conducted by Yue *et al*., focused on the injury of diabetes-related corpus cavernosum smooth muscle cell (CCSMC) associated with diabetes. The results showed that IR-61 could alleviate cellular and MitoSOX accumulation (**Figure 7G**), restore the protein levels of SIRT1 and SIRT3, and upregulate the expression of Nrf2 and HO-1 (**Figure 7H-I**), thereby reducing mitochondrial damage 126, 127. Zheng *et al*. further confirmed in the radiation-induced RILI model that IR-61 alleviates oxidative stress and improves RILI by upregulating Nrf2 and its downstream antioxidant enzyme HO-1 in lung tissues. Specifically, IR-61 not only reduces inflammatory cell infiltration and IL-1β, IL-6, TNF-α, but also decreases fibrosis factors such as collagen I/III, α-SMA, and fibronectin. It is worth noting that IR-61 can also be enriched in the mitochondria of macrophages in irradiated lung tissue, ultimately achieving continuous protection through antioxidant/anti-inflammatory/antifibrotic 30. IR-780, belonging to the same family of cyanine dyes, also exerts a strong cell protective effect through a similar oxidative stress regulation mechanism. Li *et al*. found that IR-780 pretreatment significantly reduces radiation-induced γ-H2AX expression in SV-HUC-1 cells, conferring cytoprotection by decreasing ROS accumulation and DSB damage; flow cytometry analysis confirmed concurrent upregulation of antioxidant enzymes (SOD1, SOD2, and CAT) 128. Therefore, the application of IR-780 before radiation has great protective potential for preventing acute urinary tract mucosal injury and long-term bladder dysfunction. Zhang *et al*. further confirmed that IR-780 can be enriched in the mitochondria of damaged brain microvascular endothelial cells, reducing total intracellular ROS and MitoSOX levels in cerebral microvascular endothelial cells, thereby counteracting radiation-induced damage via oxidative stress modulation 129. Therefore, this dye has the potential to protect neurovascular units and repair the BBB. Wu *et al*. further reported that IR-780 can specifically target and enrich in long-acting hematopoietic stem cells with high mitochondrial membrane potential. By inducing cells to enter a resting state, IR-780 triggers an endogenous self-protection mechanism that not only significantly reduces ROS levels and the risk of DNA damage, but also effectively enhances the regenerative and hematopoietic recovery potential of stem cells. Based on this characteristic, applying IR-780 to preprocessing strategies is expected to become an efficient intervention method in the field of stem cell protection and radiation protection 22.

In conclusion, the mitochondria-targeted TCDs have good cellular protective effects under various oxidative stress-related pathological conditions, and their mechanism is mainly related to the activation of Nrf2 mediated antioxidant defense pathways. The research results confirm that such dyes not only potently reduce cellular and mitochondrial ROS levels and alleviate DNA damage, but also upregulate the expression of various antioxidant enzymes. In clinical models such as MSC protection, myocardial infarction, diabetes-related injuries and RII, TCDs consistently reverse cell death, reduce lesion size, and accelerate tissue repair, thus emerging as highly promising therapeutic candidates for oxidative stress-related diseases.

### 4.2 Antifibrotic Effects Mediated by Glycolysis

**Figure 8:**
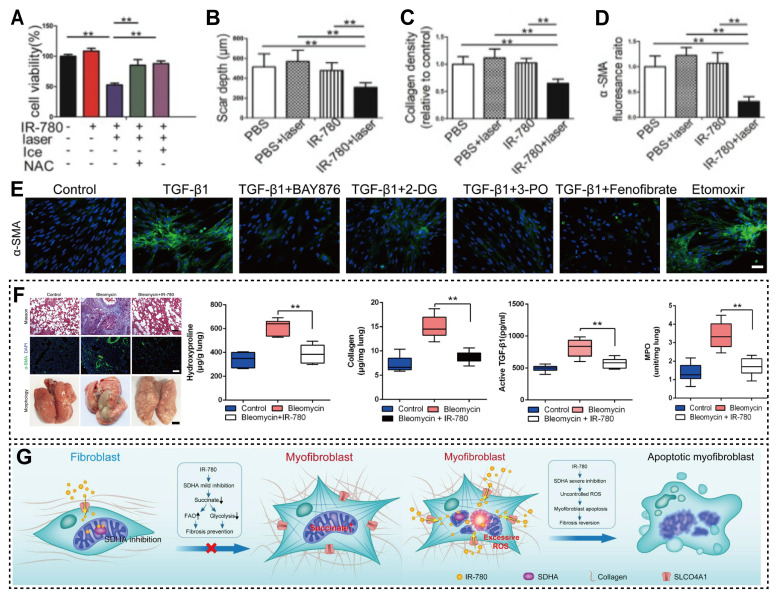
(A) Cell viability of primary fibroblasts treated with IR-780 under different treatment conditions. Analysis of scar depth (B), ECM deposition (C) and α-SMA (D) expression in healing wound tissues treated with IR-780. Adapted with permission from 27. Copyright 2019, Ivyspring International Publisher. (E) Images and their quantification results of primary lung fibroblasts treated with different treatment groups. (F) Gross morphology and related indicators of the lungs of rats after 8 weeks of treatment. (G) Schematic diagram of the mechanism by which IR-780 controls lung fibrosis. Adapted with permission from 98. Copyright 2019, Elsevier.

Tissue fibrosis constitutes a hallmark pathological feature of multiple diseases, including skin scarring, organ fibrosis, and tumor stroma formation, with the aberrant activation and proliferation of fibroblasts acting as the principal drivers of disease progression 130-132. Fibroblasts can undergo metabolic reprogramming during fibrosis, exhibiting active aerobic glycolysis, which provides a basis for targeted interventions 133-135. By utilizing the bias of TCDs towards metabolically abnormal cells, our team found that IR-780 can label glycolytic fibroblasts. Combined with directed laser irradiation, this type of pathological cells can be selectively killed. *In vitro* experiments have also confirmed that the mortality rate of IR-780 labeled cells is significantly higher than that of unlabeled controls. Calcein-AM/PI staining confirmed that the combination of PDT and PTT synergistically enhanced the photoinduced cytotoxicity of IR-780. The cell viability in the PDT-PTT combination group reached 52.6%, which was far lower than that in the PTT-alone group (85.2%) and the PDT-alone group (87.8%) (**Figure 8A**). *In vivo* experiments further verified that this phototherapy regimen can significantly reduce scar depth, extracellular matrix (ECM) deposition, and α-smooth muscle actin (α-SMA) expression (**Figure 8B-D**), thereby providing a novel approach for the intervention of excessive connective tissue deposition associated with wound healing 27. Existing studies have demonstrated that radiation induces the activation of myofibroblasts, triggers an excessive tissue repair process, leads to ECM protein deposition, and thereby results in pulmonary fibrosis 136-138. Furthermore, alveolar macrophages promote the transdifferentiation of fibroblasts and epithelial cells into myofibroblast-like cells, contributing to the progression of radiation-induced pulmonary fibrosis (RIPF) 139-141. Therefore, Luo *et al*. found that alveolar macrophages can also serve as therapeutic targets for pulmonary fibrosis. Treatment with IR-780 downregulates the expression levels of pro-fibrotic mRNAs in post-irradiation alveolar macrophages and the glycolysis inhibitor 2-deoxy-D-glucose also inhibits fibrosis development *in vitro* experiments, indicating that the antifibrotic effect of IR-780 is closely related to glycolysis regulation 142. Wang *et al*. expanded the understanding of metabolic regulatory mechanisms, demonstrating that downregulation of fatty acid oxidation (FAO) and upregulation of glycolysis in fibroblasts jointly promote pulmonary fibrosis development. They further clarified that preferential inhibition of glycolysis provides greater advantages than FAO enhancement in antifibrotic therapy (**Figure 8E**). Additionally, they showed that IR-780 can block fibroblast transdifferentiation into myofibroblasts by preventing succinate accumulation and inhibiting HIF-1α activation. To explore the potential of IR-780, Wang *et al*. treated primary fibroblasts with transforming growth factor β1 (TGF-β1) and found that IR-780 significantly upregulated mRNA and protein levels of FAO-related molecules while simultaneously downregulating key factors in the glycolysis pathway. In rat models of bleomycin-induced pulmonary fibrosis, intraperitoneal injection of IR-780 improved lung tissue morphology, reduced lung collapse and fibrous nodules, and markedly decreased expression of fibrosis-related genes, including *TGF-β1*, collagen type I alpha 1 chain (*COL1A1*), connective tissue growth factor, matrix metalloproteinase 2, α-SMA, fibronectin, and plasminogen activator inhibitor 1. After an 8-week treatment course, IR-780 was shown to ameliorate established fibrotic lesions by selectively inducing myofibroblast apoptosis (**Figure 8F**) 98. Based on previous studies, the preventive and therapeutic mechanisms of IR-780 in intervening bleomycin-induced pulmonary fibrosis have been clarified, namely that it moderately inhibits succinate dehydrogenase complex flavoprotein subunit A (*SDHA*) in normal fibroblasts and blocks the TGF-β1-mediated elevation of succinate dehydrogenase activity and succinate levels to prevent fibrosis formation and respiratory dysfunction, while potently inhibiting *SDHA* in myofibroblasts, triggering massive ROS production and further selectively inducing their apoptosis, ultimately achieving the amelioration of pulmonary fibrosis (**Figure 8G**) 98.

Hypertrophic scar (HS) is the most common complication following burns and trauma, and its core pathological feature is excessive fibrosis caused by aberrantly activated fibroblasts 143, 144. Dysregulated glycolysis has been identified as a potential therapeutic target 133, 145. Meng *et al*. demonstrated that the majority of α-SMA-positive fibroblasts in keloids exhibit hyperglycolytic characteristics, and fibroblast activation is closely correlated with enhanced glycolysis. Further experiments confirmed that IR-780 treatment can significantly downregulate the mRNA and protein expression levels of fibrosis-related factors, including α-SMA, *COL1A1*, and fibronectin in hypertrophic scar fibroblasts (HFs) and keloid fibroblasts (KFs), verifying that IR-780 inhibits the fibrotic activity of KFs by modulating the glycolysis pathway 96. Increased abundance of CAFs is often associated with a poor prognosis, tissue stiffening, alterations in the immune-tolerant microenvironment, and other adverse events 146-148. Taking advantage of the property of IR-780 to target fibrogenic fibroblasts, Yang *et al*. demonstrated that IR-780 could inhibit collagen secretion and induce CAF apoptosis. *In vivo* studies revealed that in EMT6 and MC38 xenograft models, IR-780 enhanced the anti-tumor efficacy of anti-PD-L1 therapy by reducing ECM protein deposition 97. Zheng *et al*. found that IR-61 treatment alleviated radiation-induced collagen deposition and reduced hydroxyproline levels in lung tissues. Importantly, IR-61 also significantly downregulated the expression of fibrosis-related factors at both the mRNA and protein levels. During the 20-week post-irradiation observation period, the levels of pro-fibrotic cytokines, including Arg1, Fizz1, YM-1, TGF-β1, and PDGF, in lung tissues of rats in the IR-61 treatment group were significantly decreased, demonstrating long-term antifibrotic activity 30.

In conclusion, TCDs have made a series of advances in the treatment of fibrotic diseases due to their core characteristic of targeting glycolysis. TCDs can play a dual role in inhibiting fibroblast activation and inducing abnormal cell apoptosis, whether it is promoting fibrosis in fibroblasts or CAFs. These various fibrosis-related diseases provide predictable targeted treatment strategies and therefore have important clinical research value.

### 4.3 Multi-modal Synergistic Anti-tumor Effects of TCDs

#### 4.3.1 Tumor Imaging and Therapy Mediated by TCDs

Functional materials with fluorescence properties have attracted increasing attention in the fields of sensing and imaging 108, 149, 150. The latest research has achieved differentiated recognition of cancer cells through surface modification strategies using fluorescent metal cluster arrays, and further expanded the application scenarios of fluorescent materials in NIR biomedical fields through the construction of small molecule coupled nanoparticles and optimization of NIR-II organic fluorescent cluster structures 151-153. TCDs, with its outstanding deep tissue penetration and low fluorescence background advantages, has emerged as a key tool for tumor molecular imaging. It offers a wide range of technical support for early tumor detection, lesion localization, and curative effect monitoring 154-156. In recent years, research on the integration of TCD diagnosis and therapy has become a leading trend in the field, and it has received extensive attention in the exploration of applications for precise tumor diagnosis and targeted intervention.

In the research of tumor imaging, NIR-27 has been proven to be capable of imaging a variety of tumor tissues, including gastric cancer MKN-45, lung cancer A549, and acute myeloid leukemia HL-60 tumor labeled with GFP 79. As a widely used NIR dye, the imaging application of ICG has also been continuously optimized. Researchers have further confirmed that ICG can be internalized by differentiated HCC, enabling fluorescent imaging of tumor lesions 157, 158. Owing to its superior optical characteristics and satisfactory biocompatibility, IR-780 has emerged as one of the most widely applied probes in bioimaging research 113. Zhang *et al.* confirmed that IR-780, as an efficient tumor targeting agent, can stably emit light for more than 20 days to achieve long-term reliable imaging 77. Its cellular uptake bypasses traditional endocytosis, mitochondrial voltage, or ATP-binding cassette transport pathways and instead depends on active energy metabolism, intact membrane potential, and normal OATP transporters 102.

**Figure 9:**
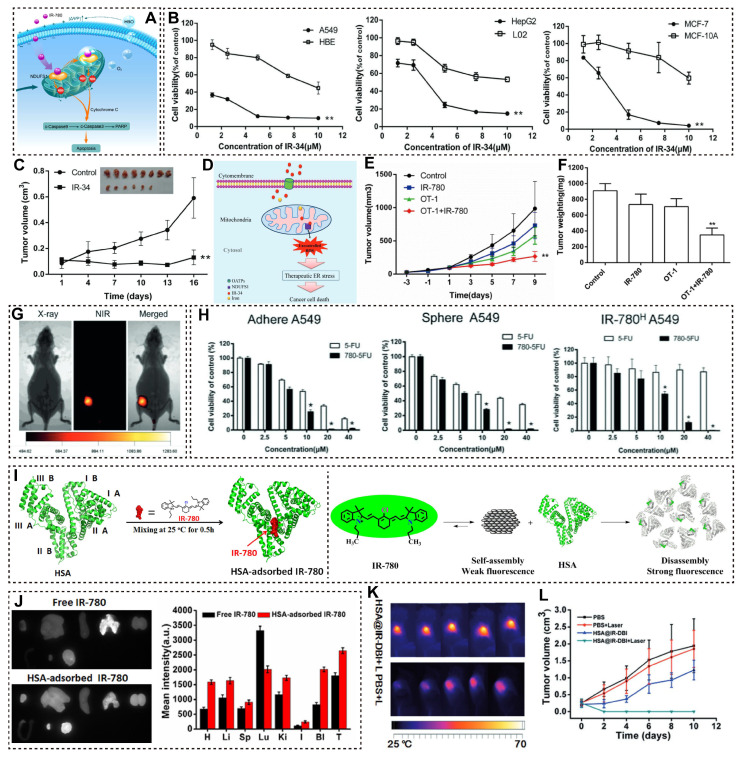
(A) Schematic diagram of the anti-tumor mechanism of IR-780 combined with hyperbaric oxygen. (B) The killing effect of IR-34 on different types of tumor cells. (C) Tumor volume change results of mice treated with IR-34. (D) The anti-tumor mechanism of IR-34. Adapted with permission from 28. Copyright 2018, John Wiley and Sons. (E-F) Tumor volume and weight of different treatment groups. Adapted with permission from 166. Copyright 2019, Frontiers Media. (G) Image results of 780-5FU in the nude mice. (H) Viability rate of cells in different states treated with 5-FU or 780-5FU. Adapted with permission from 114. Copyright 2018, John Wiley and Sons. (I) Formation process of the IR-780/HSA complex and its fluorescence enhancement mechanism. (J) Imaging and quantitative results of anatomical organs in different groups of mice. Adapted with permission from 17. Copyright 2022, BLACKWELL PUBLISHING INC. (K) Infrared thermal imaging results of tumor-bearing mice in the PBS and HSA@IR-DBI group. (L) Tumor volume results of different groups. Adapted with permission from 53. Copyright 2017, John Wiley and Sons.

Mitochondria, as the energy center that dominates cell death, are the main targets of TCDs for light-controlled signal intervention and tumor imaging 159-161. Mechanism studies have shown that IR-780 can target the mitochondria of drug-resistant A549/DR cells, triggering apoptosis by enriching ROS and collapsing membrane potential 162. IR-58, which has tumor selective toxicity, activates the TIM44-SOD2-ROS-mTOR axis, leading to excessive autophagy and programmed cell death 101. Additionally, IR-37 exerts highly efficient killing of human colorectal cancer cells via the mitochondrial apoptotic pathway. In the *in vivo* HT-29 xenograft model in nude mice, tumor growth was significantly inhibited in the IR-37 treatment group. Subsequent mechanistic studies confirmed that the SOD1-ROS-JNK pathway, regulated by the positive cofactor 4, plays a central role in the apoptotic process 163. Shen *et al*. first demonstrated that IR-780 interferes with electron transport by targeting mitochondria, leading to massive ROS production; when combined with hyperbaric oxygen, ROS further reduces mitochondrial membrane potential and exacerbates mitochondrial damage through a synergistic effect of oxygen (**Figure 9A**) 164.

TCDs are capable of eliciting anti-tumor effects by regulating ER stress or key signaling pathways. Wang *et al*. developed the probe IR-34, which can significantly inhibit the viability of various human tumor cell lines, including A549, HepG2, and MCF-7 (**Figure 9B**). *In vivo* experiments, intraperitoneal injection of IR-34 (three times per week) was shown to restrain tumor progression and postpone tumor recurrence (**Figure 9C**). Mechanistic studies revealed that IR-34 triggers tumor cell apoptosis by activating ER stress (**Figure 9D**) 28. In addition to these mechanisms, the potential of TCDs to generate synergistic outcomes in tumor immunotherapy has drawn growing research interest. Meanwhile, increasing efforts have been devoted to exploring the capacity of IR-780 for ICD induction 165. Related research by Jiang *et al*. validated that IR-780 can target mitochondria to destroy cancer cells, subsequently exposing tumor-associated antigens. This process eventually initiates ICD both *in vitro* and *in vivo*, and effectively inhibits tumor growth and metastasis. In the B16F10-OVA mouse model, the combined therapy of IR-780 and OT-1 T cells can significantly enhance the efficacy of adoptive T cell therapy (**Figure 9E-F**) 166.

Faced with the clinical challenges of strong drug resistance and high metastatic potential of melanoma, TCDs have achieved pivotal breakthroughs by virtue of their precise targeting and regulatory mechanisms. Sun *et al*. reported that IR-817 possesses strong anti-tumor potency toward typical melanoma cell lines such as A375 and B16-F10. Related mechanism analyses verified that IR-817 specifically targets the transcription factor E2F8. By regulating the E2F/Cyclin/CDK pathway, this compound triggers G0/G1 cell cycle arrest in tumor cells, and exhibits satisfactory anti-melanoma effects in both zebrafish and B16-F10 xenograft models 18. Besides IR-817, ZWZ-3 inhibits the viability of B16 and A375 cells in a concentration and time-dependent manner, with IC₅₀ values of 0.2 μM and 0.43 μM, respectively. Experimental evidence further shows that ZWZ-3 activates the mitochondrial apoptotic pathway by inducing intracellular accumulation of ROS. *In vivo* experiments showed that its tumor growth inhibition rate reached 76.3%, without significant body weight loss, thus verifying its potent efficacy and low toxicity 31. Moreover, IR-418 can selectively enrich within melanoma cells and markedly restrict tumor cell proliferation *in vitro*. Molecular mechanistic analysis indicated that IR-418 suppresses melanoma growth via activation of the Bax/Bcl-2/cleaved caspase-mediated mitochondrial apoptosis pathway. Meanwhile, it restrains melanoma metastasis by downregulating mitochondrial fission through the ERK/DRP1 signaling cascade 75. IR-545 further expands the diversity of mechanisms of action. It can not only activate the mitochondrial-mediated endogenous apoptosis pathway by reducing the mitochondrial membrane potential, but also specifically inhibit the abnormal activation of PI3K/AKT/mTOR signaling pathway 74. Notably, IR-546 plays an anti-tumor role by specifically inhibiting the transduction of the AKT/GSK3β signaling pathway 73.

Although TCDs have achieved satisfactory results in the accurate diagnosis and treatment of tumors, they still have some shortcomings, including insufficient photostability, low PCE, and low biocompatibility. In order to solve this problem, Zhao *et al*. successfully synthesized the derivative T780T by introducing the TPE between two IR-780 molecules. The PCE of T780T reached 38.5%, which was significantly higher than that of free IR-780. *In vivo* experiments confirmed that after injecting T780T solution into the tail vein of 4T1 tumor-bearing mice, the NIR laser irradiation of the tumor site for 8 minutes could elevate the local temperature to over 52 °C, which confirmed that T780T had an excellent photothermal therapeutic effect 61.

#### 4.3.2 Therapeutic Agent Delivery Mediated by TCDs

Endowed with tumor-targeting ability and biocompatibility, TCDs have emerged as ideal delivery carriers for therapeutic agents, such as chemotherapeutic drugs and radiosensitizers. Through the integrated design of targeted delivery, efficacy enhancement, and imaging guidance, these dyes effectively address the limitations of traditional therapeutic agents, including poor specificity, strong side effects, and insufficient bioavailability, thus significantly improving the precision and effectiveness of anti-tumor therapy. Jason Boyang Wu *et al*. confirmed that IR-783 can cross the BBB/BTB to accumulate in intracranial brain tumors and pituitary adenoma stem cell-like xenografts in mice. Based on this property, the researchers synthesized NIRG by conjugating IR-783 with gemcitabine. This conjugate exhibited a 7.9-fold higher accumulation in tumors than in other organs, and significantly inhibited tumor growth and metastasis 59. IR-780 and nitrogen mustard conjugate IR-780NM have stronger anti-tumor potential 102. To overcome the off-target toxicity and low targeting defects of 5-fluorouracil (5-FU) 167, Ang *et al.* linked 5-FU with IR-780 to construct 780-5FU. This fusion molecule can efficiently accumulate in the lesions of A549 tumor-bearing mice, and its anti-cancer effect is significantly better than using 5-FU alone (**Figure 9G, H**) 114.

Based on DZ-1 tumor targeting scaffolds, Zhang *et al*. and Qin *et al*. respectively developed NIRG and Abi-DZ-1 with dual effects of tumor specific imaging and growth inhibition/drug resistance reversal by coupling gemcitabine or abiraterone 51, 78. Similarly, in response to the low solubility and bioavailability of the anticancer drug s-trans, trans-farnesylthiosalicylic acid (FTS), Zhao *et al*. connected it to MHI-148 to construct FTS-148. This hybrid molecule has excellent tumor selectivity, shows stronger antiproliferative activity than free FTS *in vivo* models of pancreatic cancer, and successfully overcomes its pharmacokinetic bottleneck 80.

In addition, TCDs can also achieve multi-modal synergistic therapy by integrating radio-sensitizing functions. Gao *et al*. incorporated 2-nitroimidazole, a classic radiosensitizer, into the IR-83 to construct a novel targeted molecule with both PTT and radio-sensitizing functions. Characterization results showed that the PCE of IR-83 reached 54.1%, which was significantly higher than that of most reported photothermal therapeutic materials. *In vitro* experiments confirmed that the tumor cell apoptosis rate in the IR-83 combined with NIR laser group was significantly increased to 62%, indicating that the combination strategy of IR-83 plus NIR laser effectively enhances X-ray-mediated tumor cell damage. *In vivo* experiments demonstrated that pretreatment with IR-83 and NIR laser significantly increased tumor cell radiosensitivity to IR 52. Owing to its excellent photothermal performance, radio-sensitizing activity, and synergistic therapeutic potential, IR-83 is a highly promising candidate for a triple synergistic targeted therapy combining radiotherapy, PDT, and PTT against hypoxic tumors.

#### 4.3.3 Anti-tumor Effects of Nanoparticles Mediated by TCDs

At present, nano-carriers have been proved to have good material advantages in many diseases. Numerous investigations have found that the assembly of nano-carriers and TCDs has potential in overcoming the inherent limitations of dyes and improving the effect of tumor targeted diagnosis and treatment. The main reason for this is that the carrier can protect TCDs in space, regulate their function and modify them in a targeted manner, so as to maximize the therapeutic effect of TCDs. For example, Long *et al*. conducted research on the interaction between TCDs and carrier HSA. The results of molecular docking showed that the binding site of IR-780 was located at the interface of IIA domain and IIB domain of HSA, and they formed a stable IR-780/HSA complex through non-covalent bonds. Experiments confirmed that the fluorescence intensity of IR-780/HSA complex was significantly better than that of free IR-780, mainly due to the fluorescence enhancement effect of IR-780 aggregate depolymerization induced by HSA (**Figure 9I**). In the 4T1 tumor-bearing mouse model, the fluorescence signal intensity of the IR-780/HSA complex at the tumor site was approximately 1.5 times that of free IR-780 (**Figure 9J**), which confirmed that the dye mediated by nano-carriers could enhance the performance of PTT 17. Similarly, Tan *et al*. injected IR-DBI into the tail vein of 4T1 tumor-bearing mice, laser irradiation elevated the local tumor temperature rapidly to 60 °C (**Figure 9K**), resulting in an excellent tumor inhibition effect (**Figure 9L**). This research again demonstrates that nano-carriers can increase the photothermal characteristics of dyes 53.

To address the issues of IR-817, such as poor water solubility, susceptible fluorescence quenching, insufficient efficacy in tumor-targeted imaging, and significant side effects, Wang *et al*. prepared BSA@IR-817 nanoparticles with a PCE of 32.6%, meeting the core performance requirements for PTT. In mouse models, the tumor growth inhibition rate of the group treated with BSA@IR-817 combined with laser irradiation exceeded 99% 20. Shen *et al*. synthesized Cy750M-C1, but its poor water solubility limited its application. Therefore, they co-assembled Cy750M-C1 with DSPE-PEG_2000_ and DSPE-PEG_2000_-FA via a two-step nanoprecipitation method to prepare Cy750M-C1-FA-NPs. Molecular mechanism studies demonstrated that Cy750M-C1 activates AMPK and promotes Drp1 dephosphorylation, mediates the translocation of Drp1 and BAX to mitochondria, induces mitochondrial fission and caspase activation, and ultimately triggers programmed apoptosis of tumor cells 65. This study indicates that nanoassembly not only improves the water solubility and tumor delivery efficiency of TCDs, but also provides a foundation for their platform based functional expansion. Furthermore, in the collaborative therapy guided by photoacoustic imaging, IR-780@MPDA exhibits high drug loading capacity, enhanced tumor accumulation ability, and NIR fluorescence/photoacoustic dual-mode imaging characteristics. Under 808 nm laser irradiation, the platform achieved PDT/PTT combination therapy and induced ICD, increasing the infiltration rate of CD3^+^ T cells to 40.6% and the proportion of CD8^+^ cytotoxic T lymphocytes to 24.4%, revealing the potential association between TCD mediated phototherapy and anti-tumor immune activation 168. In addition, a representative study, researchers screened molecular level precise Cy5 core polylysine G5 dendrimers with different peripheral amino acids to evaluate their ability to induce endocytosis, efflux, and cross cellular transport. Optimized nanodots can actively deliver therapeutic antibodies into solid tumors, enhance tumor penetration, and improve immune checkpoint blockade efficacy 169. This suggests that through surface engineering and tree like macromolecular structure design, TCDs related platforms can not only achieve imaging and phototherapy, but also further undertake the active delivery function of macromolecular drugs.

#### 4.3.4 Fluorescent Dendrimers as Complementary Platforms

In addition to small molecule TCDs, fluorescent dendritic macromolecules are an important class of macromolecular platforms that can further enhance the comprehensive diagnostic and therapeutic functions of cyanine dyes 170. Dendritic macromolecules have highly branched structures, abundant terminal functional groups, and internal cavities, which can simultaneously load fluorescent dyes, targeted ligands, therapeutic drugs, and multimodal imaging components 171. For integrated diagnostic and therapeutic formulations based on cyanines, coupling or encapsulation of dendritic macromolecules can enhance water solubility, photostability, local drug loading, *in vivo* circulation behavior, and multivalent targeting ability 172. For example, studies have reported that dendritic nanoprobes labeled with Cy3/Cy5 can improve fluorescence brightness, photostability, and imaging localization accuracy, while nanohybrid materials based on dendritic macromolecules have been applied in molecular imaging, PDT, PTT, and integrated cancer diagnosis and treatment research fields 173. Therefore, fluorescent dendritic macromolecules should be considered as complementary functional amplification platforms rather than substitutes for TCDs. Future research can combine the structural targeting ability of TCDs with the multivalent functionalization achieved by dendritic macromolecules to construct a hierarchical small molecule macromolecule hybrid diagnosis and treatment integrated system. However, its synthesis complexity, size dependent clearance properties, potential cationic toxicity, and potential impact on transporter mediated cellular uptake processes all require careful evaluation 174.

In summary, TCDs have the advantage of hierarchical targeting across tissues, cells, and subcellular levels. This enables them to show excellent adaptability in a variety of scenarios, especially with strong therapeutic potential in disease intervention. The above cases confirmed that TCDs performed well in antioxidation, cell protection, antifibrosis, and anti-tumor treatment. The main reason is closely related to its synthetic modification strategy and targeting mechanism. From the design of the structure to the assignment of targeting properties, the therapeutic effect has been clearly confirmed. In addition, the dendritic macromolecule platform can further enhance the water solubility, stability, targeting affinity, and theranostic integration ability of TCDs through modular modification and multivalent targeting, providing new functional amplification strategies for their clinical translation.

## 5. Summary and Outlook

With the in-depth advancement of the precision medicine concept, the development of novel molecular tools integrating high targeting capability and multi-modal theranostic functions has become an important research direction in the biomedical field. TCDs offer broad application prospects in disease diagnosis and therapy, owing to their excellent optical properties, tunable structural features, and favorable biocompatibility. This review systematically summarizes the design strategies, targeting and molecular mechanisms, and therapeutic applications of TCDs, highlighting their advantages in integrated diagnosis and treatment. Given the practical bottleneck of transitioning from basic research to clinical translation, future research needs to focus on tackling the following directions:

(1) Precision and intelligence guided molecular design: Introducing machine learning and quantitative SARs models in the early stages of development has become the mainstream trend for precise design of dye molecules, addressing application bottlenecks such as metabolic uncertainty and unexpected immune reactions. This type of calculation method can predict the liver and kidney clearance pathways and protein crown mediated immune risk in advance, thereby selectively screening candidates for *in vivo* trait optimization 175, 176. In the field of stimulus responsive dyes, research is advancing towards finer logic control. The AND logic gate probe needs to simultaneously meet multiple microenvironment characteristics such as pH abnormality, specific enzyme expression, and tissue hypoxia in order to be activated. This “non lesion silencing” mechanism significantly reduces off-target effects and improves diagnostic safety 177-179.

(2) Photobleaching and photostability limitations: The photobleaching of TCD methylene chains under continuous laser irradiation is prone to fluorescence attenuation, which is the underlying bottleneck restricting their long-term imaging and phototherapy 180, 181. This photodamage not only deteriorates the imaging quality, but also shortens the effective treatment window by inhibiting the sustained release of ROS and decreasing the PCE 182. To this end, researchers have widely introduced modification methods such as cyanine structure modification, nano/supramolecular assembly, aggregation control, and dynamic fluorescence buffering 183-187, thus establishing a key path for building a highly stable integrated platform for diagnosis and treatment.

(3) Clinical translation and systems biology evaluation: The ultimate goal of bridging the barriers between pharmaceutical research and clinical application is to push TCDs to the forefront of clinical practice, including intraoperative tumor localization, capture of small residual lesions, and personalized treatment. However, this process is constrained by multiple factors, such as incomplete drug characteristics (metabolism and off-target accumulation), highly unreliable human EPR effects, and heterogeneity in the expression of transporters such as OATP. Therefore, there is an urgent need for bidirectional efforts in future research: on the one hand, introducing active/stimulus response mechanisms at the chemical source to deconstruct passive delivery defects, and on the other hand, conducting rigorous ADME/Tox evaluations at the biological end using physiological related models such as organoids and humanized mice to obtain reliable toxicological data 188, 189.

(4) Integration of multi-modal theranostic technologies: In response to the inherent limitations of deep tissue penetration in TCD imaging, positron emission isotopes are used to directly radio-label the stent without chelating agents, ensuring highly synchronized biological distribution and clearance dynamics between optical and PET signals 190, 191. In terms of treatment, overcoming tumor drug resistance and immune evasion is currently the core of clinical practice. Reasonably designed TCD mediated PDT can reverse the immune “cold” tumor state by triggering ICD cascade reactions to release tumor associated antigens and reshape the immunosuppressive microenvironment, resulting in significant synergistic effects with immune checkpoint inhibitors 188, 192.

(5) Expansion of disease application scope: Relying on its unique photophysical properties, the application of TCDs is extending from oncology to dynamic physiological monitoring of non-malignant lesions. Overcoming heterogeneous biological barriers is key to deepening this field: for neurodegenerative diseases, precise regulation of scaffold lipophilicity, topological polarity surface area, and molecular weight can efficiently penetrate the BBB, enabling specific detection of early β-amyloid or tau protein aggregates. In fibrosis and metabolic disease models, rationally designed TCDs focus on identifying fibroblast activation proteins or quantifying local ROS fluctuations 193, 194. Ultimately, such precise structural customization empowers TCDs to deliver quantifiable, molecular-level diagnostics for chronic conditions that routinely evade detection by conventional imaging.

In summary, TCDs, as a promising class of integrated diagnostic and therapeutic molecular tools, have demonstrated outstanding performance and potential. In future research, the relationship between structure and function should be continuously explored, and *in vitro* and *in vivo* evaluation systems should be improved. Only in this way can TCDs have the potential to further improve the effectiveness of clinical diagnosis and treatment. Ultimately, providing innovative solutions for personalized diagnosis and treatment of human diseases.

## Figures and Tables

**Table 1:** Representative TCDs classified by disease category and hierarchical targeting features.

Disease	TCDs	Structural features	Targeting mechanism	Hierarchical target	Off-target effects / selectivity limitations	Theranostic role	Biosafety	Ref
Tumor	Ph790H, DZ-1, Abi-DZ-1, FTS-148, IR-DBI, NIR-27, IR-34, IR-37, IR-58, IR-83, IR-783, IR-808DB, IR-808, IR-817, IR-780	Heterocyclic modification, Sulfonate, Carboxyl ester, Amino acid, Choline, Alkyl, 2-nitroimidazole, Abiraterone- or FTS-conjugated side chains	Mainly HIF-1α/OATP-mediated uptake; IR-780: OATP1B3 reported; DZ-1 in HCC: *OATP3A1/OATP4A1* implicated; mitochondrial membrane potential contributes to subcellular retention	Tumor tissue/Brain tumor/ GL261 tumor /Bladder tumor/ CSCs/CAFs → Cancer cells/Cancer stem-like cells or Stromal cells → Mitochondria/ Lysosomes/ER	OATP expression differs among tumor types and patients; possible uptake in normal OATP-expressing tissues; OATP subtype often not fully clarified	NIR tumor imaging, Tumor-targeted diagnosis, PTT/PDT, Drug delivery, Radiotherapy assistance, Immunotherapy, Tumor recurrence inhibition	Some dyes showed clearance from vital organs within 48 h; IR-783 showed long tumor retention; IR-34 showed two-phase half-life parameters	28, 40, 44, 46, 51-53, 59, 61, 101, 112, 77-80, 114, 163, 164, 166, 146
Fibrotic diseases	IR-780	Alkyl side chain	Glycolysis-dependent uptake; OATP subtype not specified; mitochondrial localization	Hypertrophic/Keloid scars/Granulation/ Lung → Fibroblasts/Activated fibroblasts → Mitochondria	Selectivity may depend on glycolytic activation state of fibroblasts; OATP subtype remains unclear	Imaging-guided PTT/PDT, Targeted fibroblast elimination, Antifibrotic intervention	—	27, 96, 98
Tumor associated fibroblasts	IR-780	Alkyl side chain	Preferential CAF uptake; mitochondrial accumulation; mechanism related to fibrotic phenotype and stromal metabolism, OATP subtype not specified	Tumor site → CAFs → Mitochondria	CAF heterogeneity may affect response; stromal-targeting selectivity needs validation across more tumor types	CAF-targeted theranostics; Tumor stroma modulation	—	97
Metabolic and inflammation diseases	IR-61	Heptamethine cyanine scaffold	Mitochondrial membrane potential-dependent accumulation; OATP subtype not reported	Visceral fat/ Bladder/Penis → Adipose tissue macrophages/Bladder smooth muscle cells or corpus cavernosum smooth muscle cells → Mitochondria	long-term metabolism and full off-target profile remain insufficiently defined	Anti-inflammatory regulation, Metabolic protection, Improvement of diabetic bladder dysfunction and erectile dysfunction	NIR fluorescence remained higher than that in other organs at 5 days in selected models	29, 132, 125, 126
Radiation- induced injuries	IR-ALN, IR-61, IR-780, IR-83	Aminophosphonate, sulfonate, Carboxybenzyl modification, Alkyl side chain or 2-nitroimidazole modification	Bone affinity and osteoclast precursor uptake for IR-ALN; mitochondrial targeting and Nrf2/HO-1 antioxidant regulation for IR-61; OATP involvement varies by dye and is often not subtype-defined	Bone/Lung/Bladder/Brain/Hematopoietic system / Tumor tissues under radiotherapy →Macrophages/Endothelial/Stem cells or tumor cells → Mitochondria	Bone-targeted retention and long-term clearance need evaluation; radiation injury models differ by organ	Protection against radiation-induced Bone loss, Lung injury, Cystitis, Brain injury, and hematopoietic injury; Radiotherapy enhancement	IR-ALN targets bone-related injury; IR-61 exerts antioxidant protection in RILI-related models	22, 30, 52, 56, 128, 129
Hematological malignancy	IR-26	Water-soluble amino acid ester side chain	Hyperactive glycolysis-dependent mitochondrial accumulation; OXPHOS inhibition; OATP subtype not reported	Leukemia cells → mitochondria	Selectivity may depend on AML metabolic state; off-target effects on other highly oxidative cells require evaluation	NIR imaging and leukemia-cell targeting	t_1/2_ = 56.43 ± 6.38 h; Cmax = 115.23 ± 6.73 g/L; Tmax = 4 ± 1.73 h	122

## Abbreviations

α-SMA: α-smooth muscle actin
ALN: alendronate sodium
AREs: antioxidant response elements
ALP: alkaline phosphatase
BBB: blood-brain barrier
BSA: bovine serum albumin
BTB: blood-tumor barrier
CAFs: cancer-associated fibroblasts
CAT: catalase
*COL1A1*: collagen type I alpha 1 chain
CSCs: cancer stem cells
D-A: donor-acceptor
DSBs: double-strand breaks
DMF: dimethylformamide
PDT: photodynamic therapy
ECM: extracellular matrix
EPR: enhanced permeability and retention
ER: endoplasmic reticulum
FAO: fatty acid oxidation
FCCP: carbonyl cyanide-4-trifluoromethoxyphenylhydrazone
FTS: s-trans, trans-farnesylthiosalicylic acid
GPX-1: glutathione peroxidase-1
HO-1: heme oxygenase-1
HCC: hepatocellular carcinoma cells
H₂O₂: hydrogen peroxide
HIF-1α: hypoxia-inducible factor-1α
HFs: hypertrophic scar fibroblasts
HSA: human serum albumin
hUCMSCs: human umbilical cord mesenchymal stem cells
ICD: immunogenic cell death
ICG: indocyanine green
IR: ionizing radiation
KFs: keloid fibroblasts
MitoSOX: mitochondrial superoxide
MSCs: mesenchymal stem cells
NIR: near-infrared
NM: nitrogen mustard
OATPs: organic anion transporting polypeptides
PCE: photothermal conversion efficiency
POCl₃: phosphorus oxychloride
PTT: photothermal therapy
RILI: radiation-induced lung injury
RIPF: radiation-induced pulmonary fibrosis
ROS: reactive oxygen species
SISE: stress-induced selective enrichment
SARs: structure-activity relationships
*SDHA*: succinate dehydrogenase complex flavoprotein subunit A
*SLCO*: solute carrier organic anion transporter polypeptide superfamily
TGF-β1: transforming growth factor β1
TCDs: targeting cyanine dyes
TPE: tetraphenylethylene
5-FU: 5-fluorouracil
